# ‘Arm brains’ (axial nerves) of Jurassic coleoids and the evolution of coleoid neuroanatomy

**DOI:** 10.1186/s13358-023-00285-3

**Published:** 2023-09-27

**Authors:** Christian Klug, René Hoffmann, Helmut Tischlinger, Dirk Fuchs, Alexander Pohle, Alison Rowe, Isabelle Rouget, Isabelle Kruta

**Affiliations:** 1 Paläontologisches Institut und Museum, Universität Zürich, Karl-Schmid-Strasse 4, 8006 Zurich, Switzerland; 2 Institute of Geology, Mineralogy, & Geophysics, Ruhr-Universität Bochum, 44801 Bochum, Germany; 3 85134 Stammham, Germany; 4 Jura-Museum Eichstätt, Willibaldsburg, 85072 Eichstätt, Germany; 5 SNSB-Bayerische Staatssammlung für Paläontologie und Geologie, Richard-Wagner-Straße 10, 80333 Munich, Germany; 6 Centre de recherche en paléontologie - Paris, Sorbonne Université-MNHN-CNRS-CR2P, 4 Pl. Jussieu, 75005 Paris, France

**Keywords:** Cephalopoda, Neuroanatomy, Nervous system, Ganglion, Brain, Konservat-Lagerstätte, Taphonomy

## Abstract

Although patchy, the fossil record of coleoids bears a wealth of information on their soft part anatomy. Here, we describe remains of the axial nerve cord from both decabrachian (*Acanthoteuthis, Belemnotheutis, Chondroteuthis*) and octobrachian (*Plesioteuthis, Proteroctopus, Vampyronassa*) coleoids from the Jurassic. We discuss some hypotheses reflecting on possible evolutionary drivers behind the neuroanatomical differentiation of the coleoid arm crown. We also propose some hypotheses on potential links between habitat depth, mode of life and the evolution of the Coleoidea.

**Supplementary Information:**

The online version contains supplementary material available at 10.1186/s13358-023-00285-3.

## Introduction

The cephalopod arm crown is a fascinating body part, which inspires both researchers and artists, movie makers and authors of fiction. In recent years, the arm crown and other decentralized functions of cephalopod bodies, such as vision and the control of skin colouration, and arm movements independent of the central nervous systems have been widely discussed. For example, discussions regarding cephalopod vision and skin colouration (camouflage) are rooted in a debate between Hess and Frisch (Frisch, 1912; Hess, 1902, 1905, 1912), which was later resolved (Dröscher, 2016; Messenger, 1977; Messenger et al., 1973), although the discussion on the presence or absence of colour vision continues (Stubbs & Stubbs, 2016). Knowing the colour-blindness of octobrachians, their camouflaging capabilities are even more surprising. Kingston et al., (2015a, 2015b) found an explanation in the discoveries of dermal photoreception and of “Eye-independent, light-activated chromatophore expansion (LACE)” (i.e. colour change independent of the eyes; see Ramirez & Oakley, 2015; Katz et al., 2021). Independent of this discussion, the question for links between the neural equipment of the arms, overall neuroanatomy, and the (palaeo-) environment arises. Such links were found by, e.g., Chung et al., (2022a, 2022b) and are discussed here for extinct coleoids.

**Fig. 1:**
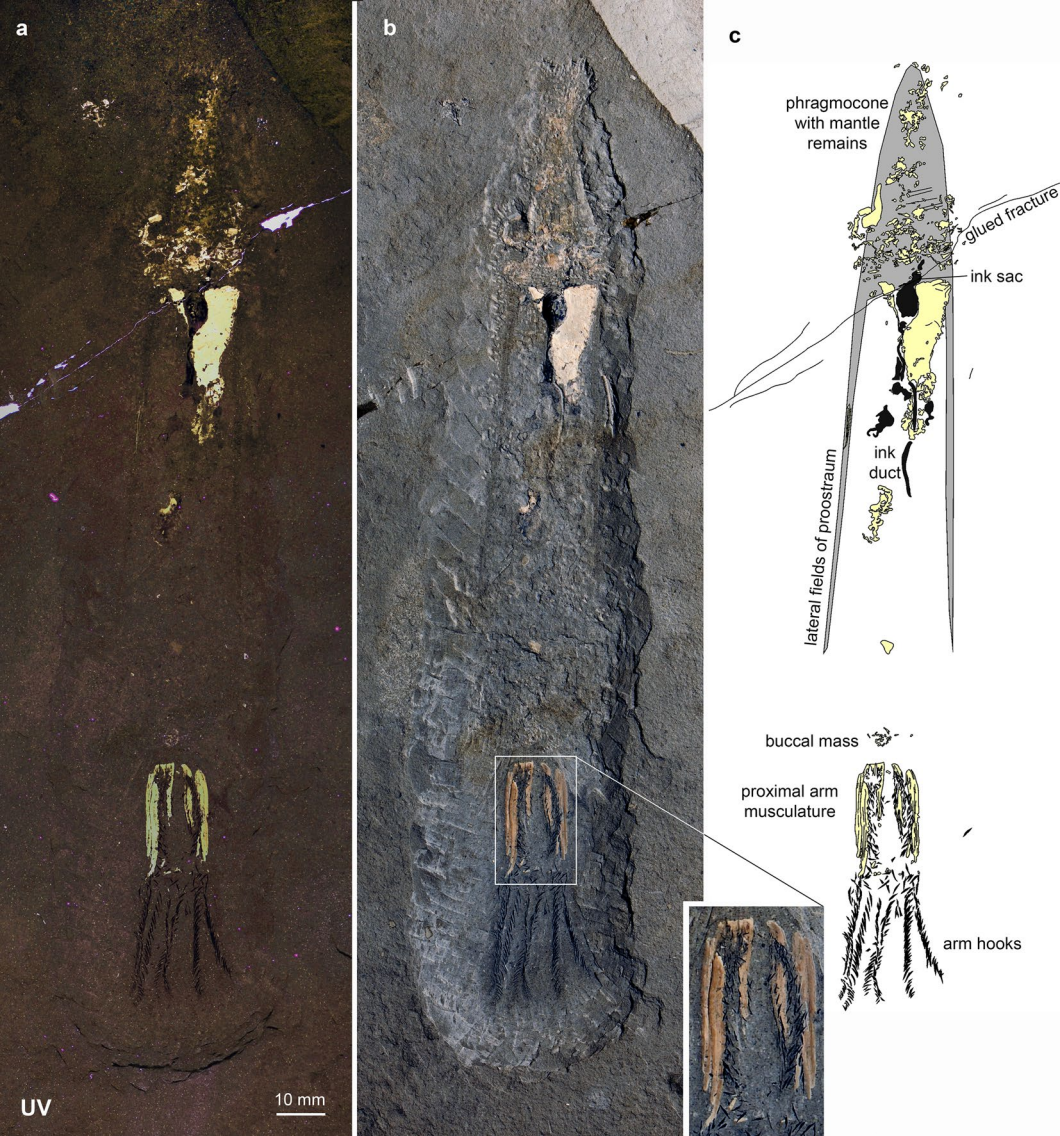
*Sueviteuthis zellensis* Reitner & Engeser, 1982, GPIT Ce 1564/2,6/PV-67025, Toarcian, Fischer quarry, Zell, east of Ohmden. **a** UV-photo by R. Roth; **b** photo under white light (insert with magnification of the arm bases); **c** drawing after a

The neural equipment of cephalopod arms is linked with the organs of the arms such as suckers (Graziadei, 1962). Suckers are interesting sensory components of the coleoid arm crown, and likely originated in the Carboniferous, or even earlier (Fuchs et al., 2010, 2021; Kröger et al., 2011; Kruta et al., 2016; Tanner et al., 2017; Whalen & Landman, 2022). More than half a century ago, Wells, (1963, 1964) documented the presence of chemosensory and tactile receptors in *Octopus* (see also Chase & Wells, 1986; Lee, 1992; Maselli et al., 2020). Refined experiments confirmed the chemoreceptors to be located within their suckers (Buresch et al., 2022). This is important in the generally dark, murky waters where they live as they use their arms to forage for prey in crevasses. Importantly, in octopods, the sensory information is processed initially by ganglia present in the suckers and then forwarded to the larger intrabrachial ganglia on the axial nerve cord (Graziadei, 1962; fig. 1; Young, 1971). Octopods have indeed a complex brachial nervous system related to their “*sophisticated use of their arm and their ability for tactile learning*” (Budelmann, 1995: p. 125).

Preservation of nervous systems is known only from fossil localities yielding exceptional preservation. The fossil record of soft tissues is best known among octobrachians (e.g., Clements et al., 2017; Fuchs et al., 2010; Fuchs, 2006a, 2006b; Klinghardt, 1932; Klug et al., 2015; Naef 1922; Rowe et al., 2022, 2023). Less is known about the soft tissues of decabrachians (Fuchs et al., 2010; Fuchs, 2006a, 2006b; Klug et al., 2016, 2019), and less still about externally shelled (ectocochleate) cephalopods such as ammonoids and nautiloids (De Baets et al., 2013; Klug & Lehmann, 2015; Klug et al., 2012, 2015, 2021a, 2021c). Some of these reports (Fuchs et al., 2010, 2021; Fuchs, 2006a, 2b; Klug et al., 2016, 2020; Kruta et al., 2016; Rowe et al., 2022, 2023) demonstrated the increased preservational potential for soft tissue anatomy in the coleoid arm crown, which are typically less common than the sclerotized elements such as arm hooks or sucker rings.

In contrast to many other soft-tissue details, descriptions of the nervous system of cephalopods have only occasionally found their way into the scientific literature (Fuchs & Larson, 2011a, 2011b; Fuchs, 2006a, 2006b; Jattiot et al., 2015; Klug et al., 2016, 2019, 2021a; Larson et al., 2010), mostly showing parts of the cephalic cartilage. Fossilized axial nerve cords that probably included the intrabrachial ganglia, the brain-like concentrations of neuronal tissues in the arms, have been documented only a few times (Kruta et al., 2016; Rowe et al., 2022, 2023) and once unknowingly (Klug et al., 2016, supplementary Fig. 5). This exceptional preservation is limited to conservation deposits (Seilacher, 1970) and, so far, have only been discovered in the German Posidonienschiefer (Toarcian, Early Jurassic, this paper), the French Callovian marls of La Boissine (La Voulte-sur-Rhône, Middle Jurassic; Kruta et al., 2016; Rowe et al., 2022, 2023) and the German platy limestones of the Solnhofen region (Late Jurassic; Klug et al., 2016). Remains of arms and axial nerve cords were also discovered in other Fossillagerstätten such as the English sites of Christian Malford (Middle Jurassic), and the Late Jurassic coastline (this paper) or the Cenomanian of Lebanon (Larson et al., 2010: figs. 3B and 5I) and, without axial nerves, in the Oligocene of Russia (Mironenko et al., 2021).

In this study, we describe some specimens showing phosphatized remains of arm soft-tissues from the Jurassic of France and Germany. We attempt a homologization of the visible structures and discuss their nature. Further, we put these structures into the evolutionary context of the origin of major coleoid clades. We assess potential links between habitat and neuroanatomy of these fossil coleoids.

## Methods

As an independent test of our evolutionary hypotheses on habitat distribution, we performed Bayesian ancestral state reconstructions (Pagel et al., 2004). For this purpose, we used the ingroup of the time-calibrated phylogeny of Tanner et al., (2017), which is based on transcriptomic data from 26 extant cephalopod species. Fossil species were not included because timetrees uniting the relevant living and fossil taxa are currently not available. Each taxon was scored for their habitat distribution following Chung and Marshall, (2017), i.e. coastal (category GI) and pelagic (categories GII–III). We did not divide pelagic cephalopods further into deep pelagic and vertical migrating species because this trait is more variable, and a wider species coverage would be needed for accurate predictions. Nevertheless, at least within this dataset, pelagic species always are taxa that can regularly be found at depths of hundreds of metres or live permanently in the deep sea, while coastal species are restricted to depths of less than 200 m, mostly within the photic zone. Thus, it is conceivable that this habitat switch has important implications for the nervous system, as adaptations to low light conditions may be essential for pelagic species regardless of whether they are vertical migrants or deep-sea dwellers.

Ancestral state reconstructions were performed in RevBayes version 1.1.1 (Höhna et al., 2016), using the Mk model with equal transition rates (Lewis, 2001) and fixed tree topology. The prior on the transition rate and only parameter of the model was set to an exponential distribution. The MCMC algorithm was run for two independent replicates with 25,000 generations using a random move schedule, discarding 25% of the samples as burn-in. The output was then processed in R using the package RevGadgets version 1.1.0 (Tribble et al., 2022). The script for the analysis in RevBayes and its output are provided in Additional File 1.

The specimen of *Proteroctopus ribeti* (MNHN.F.R03801) and *Vampyronassa rhodanica* (MNHN.F.B74244) originally described by Fischer and Riou (1982, 2002), were reanalysed using propagation phase contrast synchrotron X-ray micro-computed tomography (PPC-SRµCT, ESRF-ID19) in Kruta et al., (2016) and Rowe et al., (2022, 2023). Acquisition details are provided in these publications. Additional PPC-SRµCT slices are illustrated here in order to show the axial nerves in the specimens. Measurements were taken only where the section of axial nerve was visible in all three views (cross section, coronal, and longitudinal).

## Results

### Descriptions

Systematics and phylogeny are according to Hoffmann et al., (2022). We follow the order provided there on page 192. Below, we provide descriptions of the head-foot with a focus on the arm crown. Since all the specimens presented here show exceptional preservation, the number and shape of arms is more or less well known. In combination with the preserved hard parts, their placement within the decabrachians and octobrachians is quite well supported (e.g., Fuchs, 2006a, 2016; Haas, 1997; Jeletzky, 1966; Kröger et al., 2011; Tanner et al., 2017).

In some specimens, remains of the brachial nervous system (e.g., the axial nerve cord) are preserved. Others retain armature from which it is possible to infer function and therefore extrapolate the complexity of the central nervous system associated with the sensory tasks.

Crown group node Neocoleoidea Haas, 1997

Superorder Decabrachia Haeckel, 1866

Order Belemnitida Haeckel, 1866

Family Belemnotheutidae Zittel, 1884

*Sueviteuthis zellensis* Reitner & Engeser, 1982

(Fig. 1)

*Specimen* GPIT Ce 1564/2,6 (GPIT-PV-67025), paratype, original of Reitner and Engeser (1982:3, fig. 4, pl. 1).

*Stratigraphy* Posidonia Shales, Koblenzer, Semicelatum Subzone, Tenuicostatum Zone, Toarcian, Early Jurassic.

*Locality* Fischer quarry, east of Ohmden, Germany.

*Description of arm crown* The entire specimen is about 190 mm long. It preserves the complete phragmocone, most of the proostracum, the ink sac and most of the ink duct, and a complete arm crown with stretched out, subparallel arms. The arm crown is 53 mm long and preserves the remains of 391 small arm hooks. They are arranged in rows, which can be assigned to at least seven arms. The stylet-shaped arm hooks are of varying size, ranging up to 3.2 mm long. The proximal parts of the hook-rows are associated with elongated phosphatized fields. These fields are up to 25 mm long, between 1 and 2 mm wide, and bear very fine longitudinal striations which likely represent musculature. Depending on how these fields are counted (because the visible separation of the fields varies strongly), there are between six and nine distinct fields. When accepting the higher number, the maximum width is around 1 mm. There appears to be a gap between the distal end of these phosphatized fields and the distal 28 mm of the arms. This gap is present in all of the visible arms, suggesting a primary structure differentiating the proximal from the distal portions. A few arm hooks are present between these fields, though the majority of aligned hooks are clustered in the distal section.

*Acanthoteuthis speciosa* Münster, 1839

(Fig. 2)

**Fig. 2:**
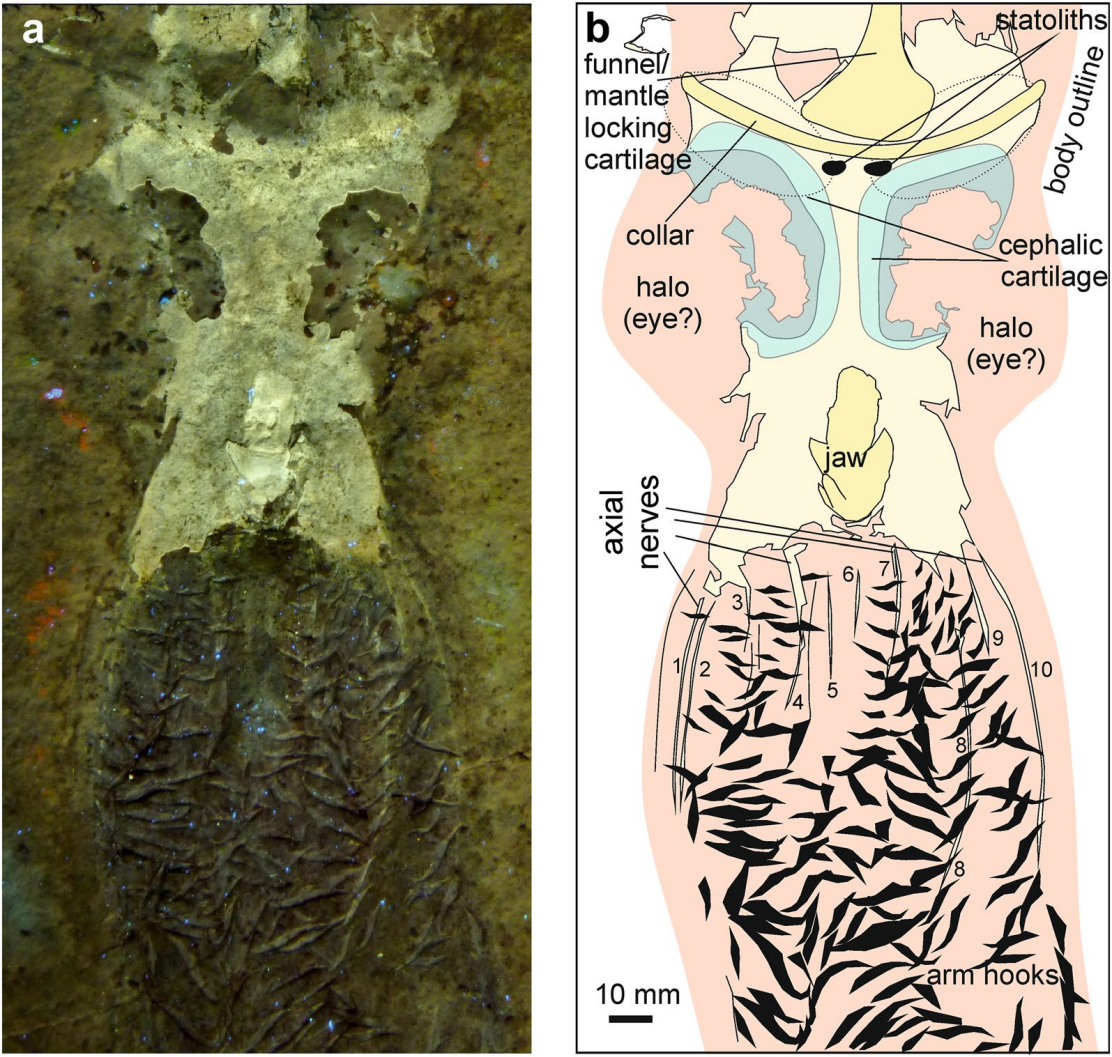
*Acanthoteuthis speciosa* Münster, 1839, HT 02/2, latest Kimmeridgian, Öchselberg Quarry near Zandt, Bavaria (Germany). Figure modified after Klug et al., (2016, suppl. Fig. 5). **a** UV-photo by HT; **b** drawing after a

*Specimen* HT 02/2, original of Klug et al., (2016, supplement, p. 8, figs. S4, S5).

*Stratigraphy* Lithographic Limestones, Beckeri-zone, Ulmense subzone, Rebouletianum horizon, uppermost Kimmeridgian.

*Locality* Öchselberg Quarry near Zandt, Bavaria (Germany), Germany.

*Description of arm crown* The entire specimen measures 440 mm from the tips of the arms to the apex of the rostrum. The head foot-complex is exceptionally preserved, displaying statocysts with statoliths (Klug et al., 2016, supplement), cephalic cartilage, body outline, jaws and the arms. The arms are up to 120 mm long and nearly 300 hooks are discernible. The arm hooks belong to the *Acanthuncus* morphotype and show the characteristic changes from the proximal to distal parts of the arm crown. Proximally, the hooks are small and almost straight, while those in the mid-section are much larger, strongly curved and have a large uncinus. Distally, they become shorter again. Between the hook rows, UV-photos (Fig. 2a) show fine phosphatized lines that run parallel to the hook rows. These lines are up to 1.5 mm wide and between 15 and about 70 mm long. Traces of about 10 such lines are visible particularly in the proximal half of the arms. Several are linked with the phosphatized surface surrounding the jaws.

*Acanthoteuthis* sp.

(Fig. 3)

**Fig. 3:**
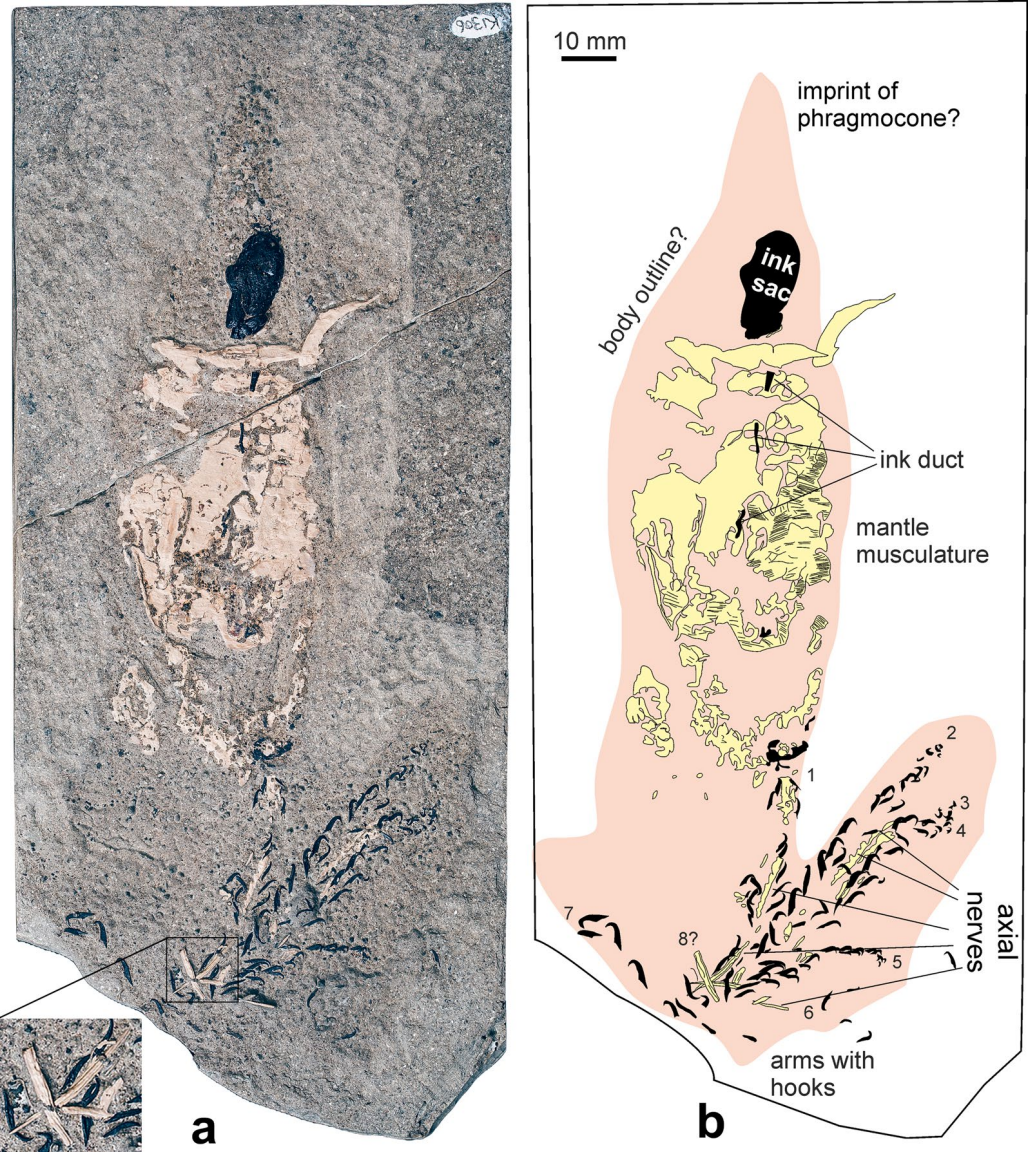
*Acanthoteuthis* sp., KI306, Etches collection, Kimmeridgian, Kimmeridge Bay (United Kingdom). Photo by Terry Keenan. **a** photo of specimen, with magnified detail to show the striation of the axial nerves. **b** Sketch of the specimen

*Specimen* KI306, Etches collection.

*Stratigraphy* Kimmeridge Clay, Kimmeridgian.

*Locality* Kimmeridge Bay, United Kingdom.

*Description of arm crown* The body outline as indicated in Fig. 3b is about 180 mm long. The specimen preserves the ink sac and duct, an imprint of the phragmocone, phosphatized remains of the mantle musculature and parts of the arm crown. Four arms (up to 60 mm long) are reasonably complete with the majority of the arm hooks being preserved. The hooks look like those of *A. speciosa* and *Belemnotheutis antiquus* (*Acanthuncus* morphotype), an issue of systematics of these taxa that will need clarification in the future. The arm hooks are up to 6 mm long and display the characteristic change in size and shape from the arm base (smaller, less curved) to the middle hooks, which are the largest and most strongly curved back to the distal ones, which are gently curved sinusoidally and small. Between the arm hooks, phosphatized longitudinal structures are discernible. These are 1–2 mm wide and up to 16 mm long. Some are longitudinally striated (Fig. 3a insert). Based on these structures and the groupings of arm hooks, we identified remains of eight of the ten arms. The remaining two arms might be missing because the adjacent slab was likely already lost when the specimen was found.

*Belemnotheutis antiquus* Pearce, 1842

(Fig. 4)

**Fig. 4:**
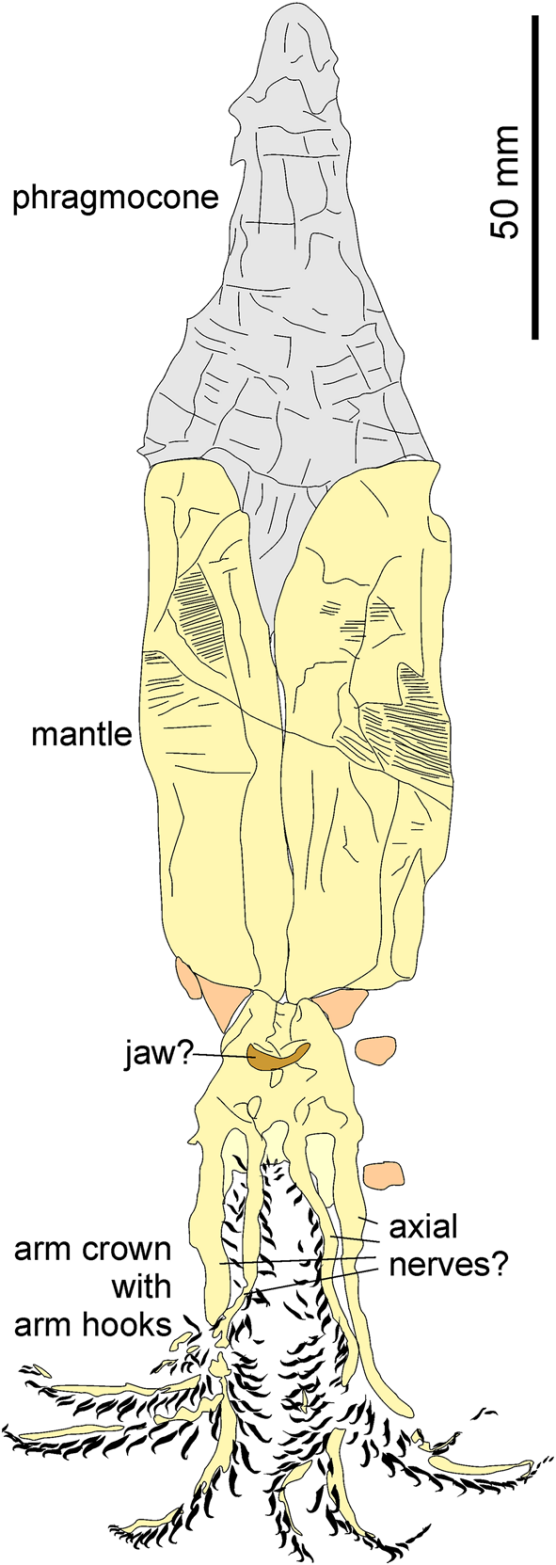
*Belemnotheutis antiquus* Pearce, 1847; NHMUK 25966, Christian Malford, Wiltshire, England, Callovian Oxford Clay; entire animal with excellently preserved mantle and complete arm crown. Drawing after the holotype, refigured in Clements et al. (2017)

*Specimen* NHMUK 25966, Natural History Museum, London; original of, e.g., Owen, (1844) and Pearce, (1847).

*Stratigraphy* Oxford Clay, Athleta Zone, upper Callovian,

*Localit*y Christian Malford, Wiltshire, United Kingdom.

*Description of arm crown* This specimen is included for its excellent preservation and historical importance. It is also remarkable because it tells a story of the classical conflict between collectors and well-informed laypersons on the one hand (such as the amateur palaeontologists Mary Anning, who first discovered belemnotheutid materials in 1826, and Joseph Pearce, who introduced the genus and species in 1842) and professionals on the other hand, sometimes arrogant like Richard Owen, (1844) in this context, or correct such as Gideon Mantell, (1848). See Donovan and Crane, (1992) for a detailed historical report and description of the taxon.

The specimen is about 243 mm long. It is complete and preserves the phragmocone (72 mm long) with the proostracum, which is largely covered by the phosphatized mantle musculature (81 mm long and 48 mm wide in its flattened state). The head region is also phosphatized but is poor in anatomical detail. The centre displays a 10 mm wide crescent-shaped structure, which is tentatively interpreted as part of the jaw. The arm crown is very well preserved and shows the remains of at least seven arms with more than 240 distinct arm hooks, ranging between 1 mm (proximally and distally) and 5 mm (about 20–30 mm from the tips) in length, showing the previously mentioned shape change. Each double row of arm hooks is accompanied by an elongate phosphatized structure, which is 1–5 mm wide. It is unclear whether these structures represent the complete arms or parts thereof. Since it is a historic specimen, it is conceivable that parts of arm width were lost due to preparation efforts (for a photo see Clements et al., 2017).

Order Diplobelida Jeletzky, 1965

*Chondroteuthis wunnenbergi* Bode, 1933

(Figs. 5, 6)

**Fig. 5:**
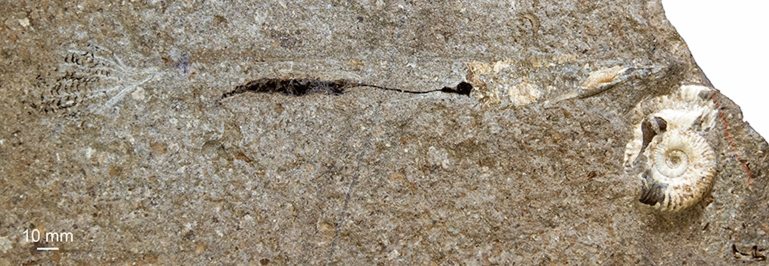
*Chondroteuthis wunnenbergi* Bode, 1933, BGR MA 13436, Hondelage near Brunswick (Germany), Toarcian Posidonia Shale, entire animal with ink sac and duct as well as phragmocone and rostrum. The ammonite may be a hammatoceratid or harpoceratid. White light

**Fig. 6:**
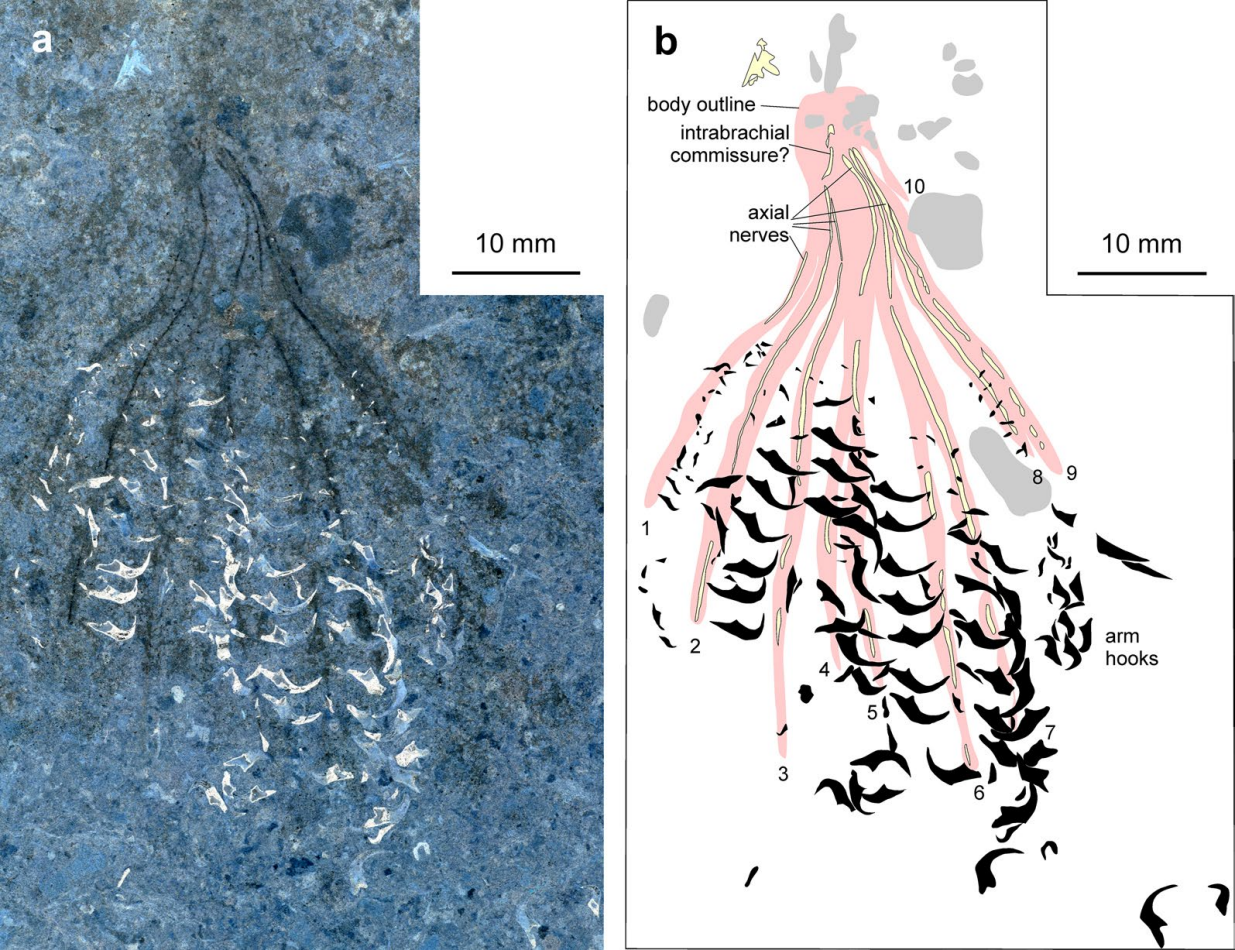
*Chondroteuthis wunnenbergi* Bode, 1933, BGR MA 13436, Hondelage near Brunswick (Germany), Toarcian Posidonia Shale. **a** Arm crown, inverted white light photo by RH; arm hooks appear whitish, the axial nerves are dark. **b** Drawing after a. Pink—outlines of arms; black—arm hooks; light yellow—lightly phosphatized structures; grey—dark spots in the sediment

*Specimen* BGR MA 13436 (described in Hoffmann et al., 2017, specimen two therein).

*Stratigraphy* Posidonia Shale, Falciferum Zone, Toarcian, Early Jurassic.

*Locality* Hondelage near Brunswick, northern Germany.

*Description of arm crown* The specimen is complete and preserves the rostrum and phragmocone remains, imprints of parts of the proostracum, the ink sac with ink duct (Fig. 5) and a complete arm crown (Fig. 6). The entire specimen measures 170 mm from the apex of the rostrum to the tip of the arms. The arms are quite slender and up to 60 mm long. About 190 arm hooks are discernible, ranging between 0.2 and 3 mm in length, with the largest hooks in the middle of the arms. Typical for this taxon, the hooks are arranged in a single row (instead of biserial rows) and belong to four different hook morphotypes (see Hoffmann et al., 2017). The distal arm hooks appear to be missing, which is likely a taphonomic artefact. The larger hooks have a broad base and a long uncinus. These are visible in the overview photograph (Fig. 5), though to enhance the contrast, another more detailed photograph was taken and inverted to obtain a negative image of the arm crown. This shows a set of pale stripes associated with the arm hook-series that likely represent faint remains of the arms. Within these faint outlines, light grey lines (Fig. 5, dark in Fig. 6a and light yellow in Fig. 6b) are visible, which can be traced from the base to the tip in some of the arms. In total, 10 arm traces could be identified, but only nine of which display the more distinct finer internal line. The arms seem aligned in two bundles. Between the bases of these two bundles, an oblique connecting line is visible (intrabrachial commissure in Fig. 6b).

Order Spirulida Haeckel, 1866

*Spirula spirula* (Linnaeus, 1758)

(Fig. 7)

**Fig. 7:**
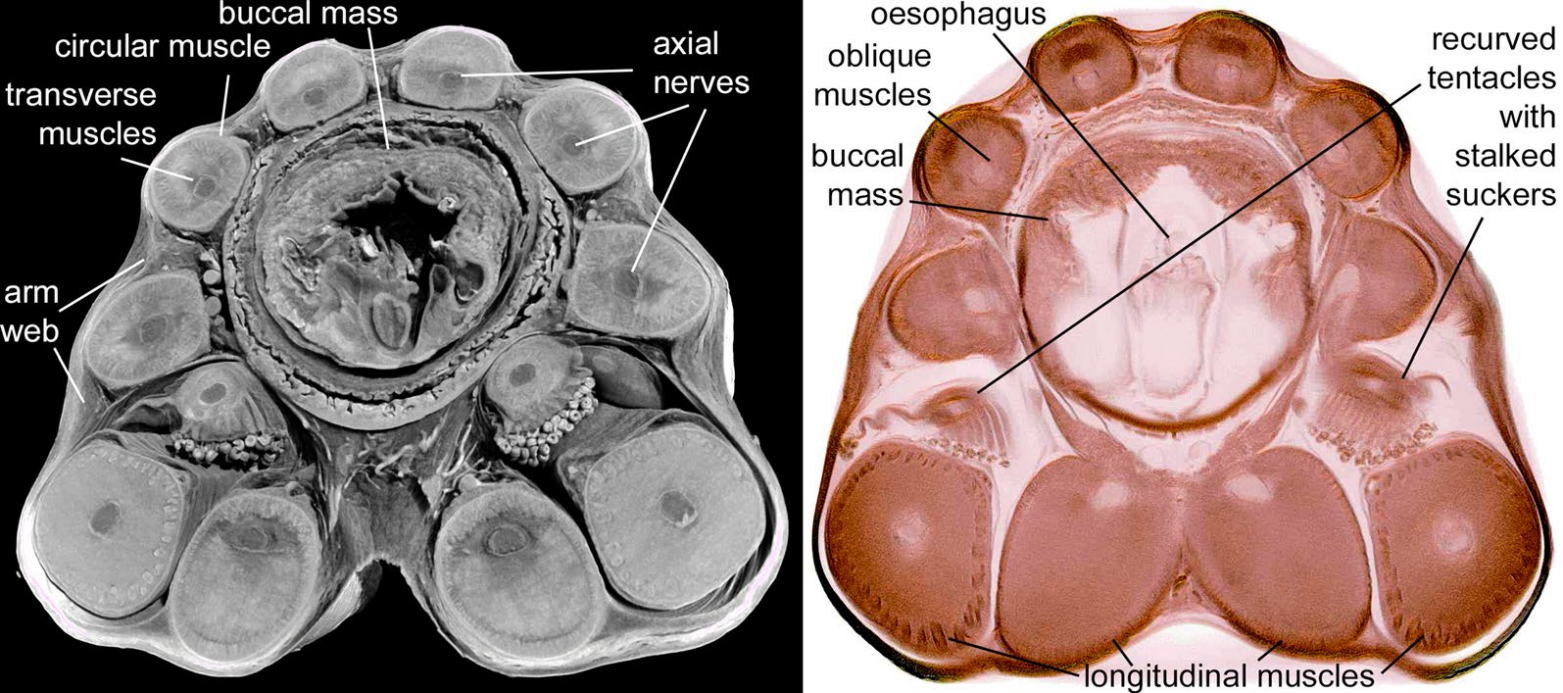
Orthoslice made from a CT-data volume (left) and histological section (right) cutting through the arm bases and buccal mass of *Spirula spirula* (Linnaeus, 1758). The arm bases encircle the buccal mass. Each arm has longitudinal muscle fibres surrounding an indistinctive grey mass. The darker spot in the central or subcentral position represents the axial nerves. The ratio between arm diameter and nerve diameter varies between 6:1 in dorsal arms and 4:1 in ventral arms

*Description* The entire specimen is about 40 mm long. For CT-scanning, the extant specimen was stained with tungsten. Due to a subsequent staining with iodine, the specimen was unfortunately destroyed. The complete arm crown was preserved and composed of eight arms and two tentacles. All of these appendages curved inwards. The visible part of the arm crown is 6 mm long. It shows small biserial suckers that extend from the base to the middle section of the arms, before merging into a single row at the distal tips. Like the hooks in other species, the suckers decrease in diameter towards the arm tips but have their largest diameter at the first third of the arm. An orthoslice view cutting through the arm bases (Fig. 7) shows the central axial nerve cords surrounded by the arms musculature or slightly moved inwards as darker spots of 0.19–0.27 mm diameter (arm diameter/nerve diameter = ratio). For a list of measurements, see Table 1. The shell has a diameter of 14 mm and 26 chambers.

**Table 1:** List of arm measurements and proportions of *Spirula spirula*

Arm	Arm diameter [mm]	Axial nerve diameter [mm]	Ratio arm/nerve diameter
1	1.61	0.25	6.44
2	1.45	0.27	5.37
3	1.18	0.21	5.62
4	1.16	0.19	6.11
5	1.22	0.22	5.55
6	1.19	0.23	5.17
7	1.19	0.25	4.76
8	1.15	0.21	5.48
9	1.41	0.27	5.22
10	1.61	0.26	6.19

Superorder Octobrachia Haeckel, 1866

Order Vampyromorpha Robson, 1929

*Remarks* Here, we briefly summarize anatomical information about the arm crown as it was obtained in the initial descriptions by Fischer and Riou, (1982, 2002) as well as the re-descriptions using synchrotron data published by Kruta et al., (2016) and by Rowe et al., (2022, 2023).

*Proteroctopus ribeti* Fischer & Riou, 1982

(Fig. 8)

**Fig. 8:**
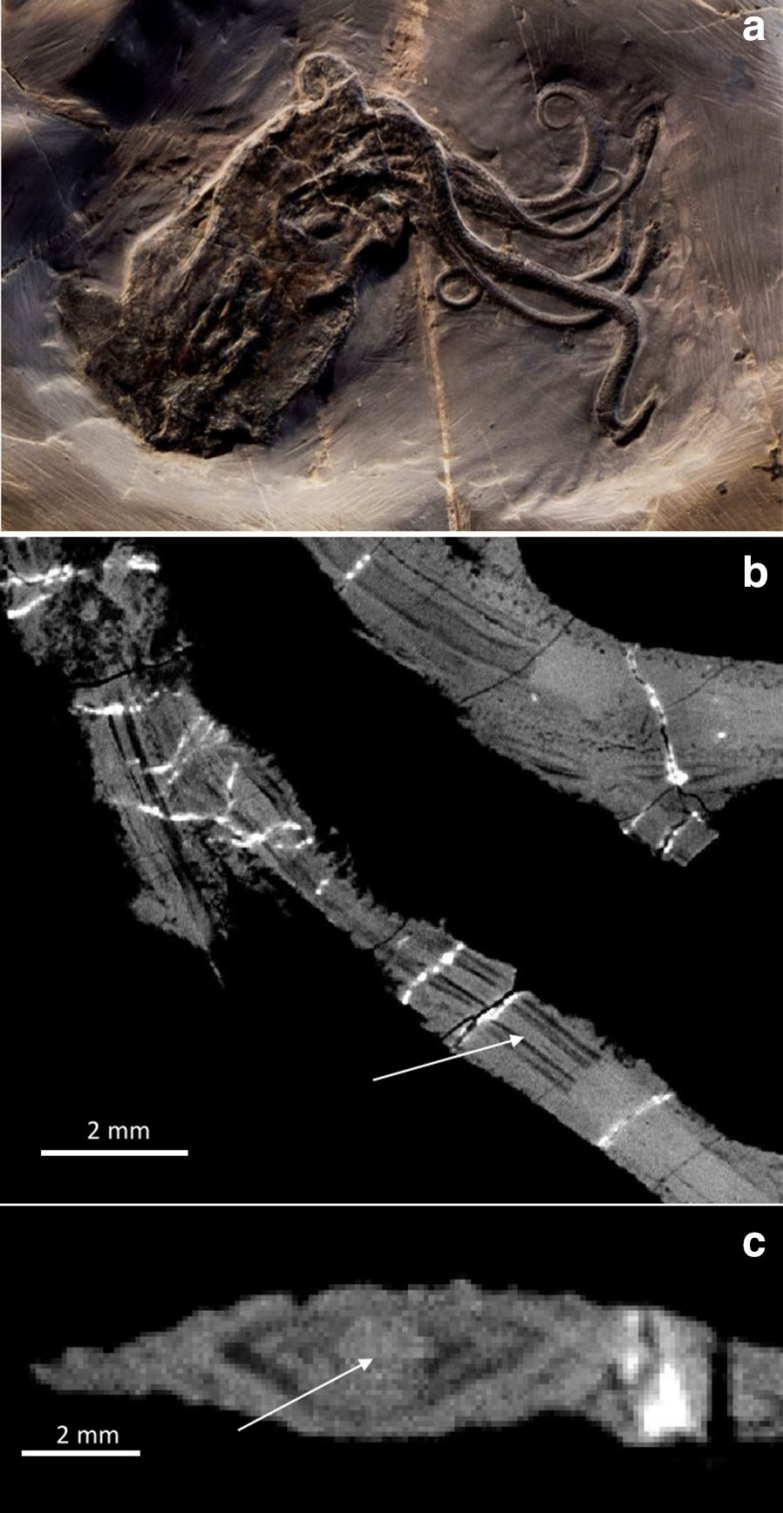
*Proteroctopus ribeti* Fischer & Riou, 1982, MNHN.F.R03801. **A** Photograph by P. Loubry, reproduced with permission, CR2P. **B** PPC-SRμCT (ESRF-ID, ID 19, voxel size 44,5 µm) slice of arm crown showing axial nerve (coronal view). **C** Cross section of the arm crown. Arrow is pointing to the axial nerve seen at the centre of the arm musculature seen in B and C

*Specimen* MNHN.F.R03801, holotype of Fischer and Riou, (1982).

*Stratigraphy* Koenigi Zone, early Callovian, Middle Jurassic.

*Locality* La Voulte-sur-Rhône, Ardèche, France.

*Description of arm crown* Like *Vampyronassa*, the holotype of *P. ribeti* is extremely well preserved, showing detail rarely seen in other coleoid fossils. The holotype measures about 120 mm in length with a 68 mm long mantle (Kruta et al., 2016: p. 2, Fig. 8a). Fins are clearly visible. The head is rather short with big eyes, though neither reveal a lot of anatomical detail. By contrast, the arm crown is complete and shows suckers and internal anatomical details. In fig. 1F of Kruta et al. (2016) and in Fig. 8b, c, the axial nerve cords are evident in the tomographic image. At an arm diameter of about 2.5 mm, the axial nerve is about 0.5 mm wide.

*Vampyronassa rhodanica* Fischer & Riou, 2002

(Fig. 9)

**Fig. 9:**
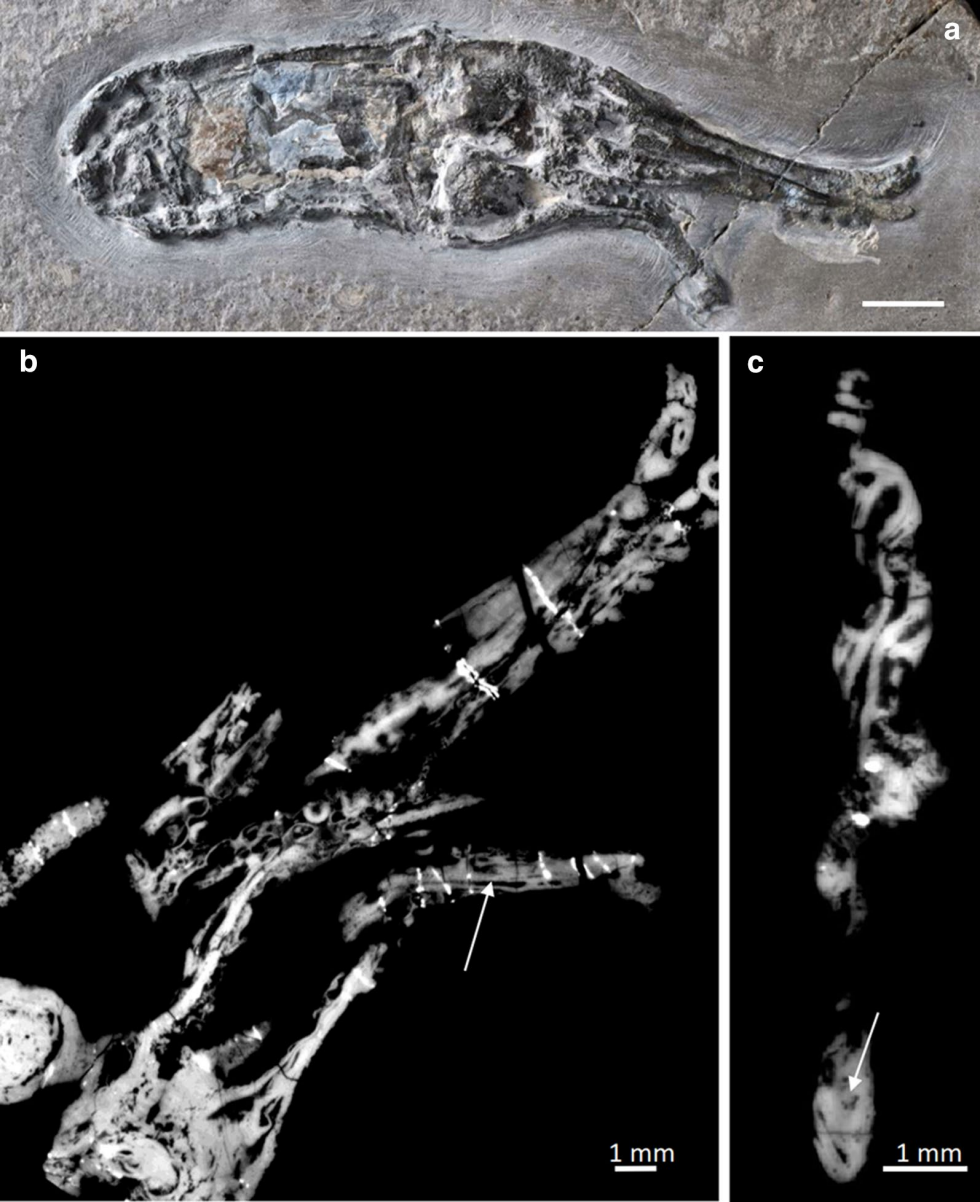
*Vampyronassa rhodanica* Fischer & Riou, 2002, MNHN.F. R03801. **A** Photograph by P. Loubry, reproduced with permission, CR2P. **B** PPC-SRμCT (ESRF, ID 19, voxel size 25 µm) slice of arm crown showing axial nerve in the centre of the arm crown (coronal view). The suckers are also visible. **C** Cross section of the arm crown. Arrow is pointing to the axial nerve seen in b

*Specimen* MNHN.F.B74244, paratype of Fischer and Riou (2002)

*Stratigraphy* Koenigi Zone, early Callovian, Middle Jurassic.

*Locality* La Voulte-sur-Rhône, Ardèche, France.

*Description of arm crown* MNHN.F.B74244 has undergone some rotation and compaction prior to fossilization, though many of the original soft tissues are preserved in 3D (Rowe et al., 2022). The overall length (posterior-most tip of the mantle to the distal tip of the dorsal arms) of the specimen measures approximately 97 mm. There is no evidence that *V. rhodanica* possessed an ink sac. The head, which is fused to the mantle, preserves both eyes. Their position and subspherical shape (about 5–7 mm in diameter) likely reflect the compaction of the specimen.

Eight arms are visible in the arm crown (Fig. 9). The preserved length of the two dorsal arms is about 43 to 51 mm. This is approximately equivalent in length to the mantle (about 46 mm). The configuration of the armature on the dorsal arms comprises two distally positioned uniserial suckers, flanked by biserial cirri. The suckers are about 2 mm in diameter.

The six non-dorsal arms are shorter and their preserved length ranges from 24 to 36 mm. Up to ten uniserial suckers are visible per arm, and range in diameter from 1.6 mm (proximally) to 0.8 mm (distally). These are continuous along the length. Biserial cirri flank these suckers. They have a similar diameter and same tapering pattern as the suckers.

The axial nerves of each of the arms are visible in the PPC-SRµCT slices Fig. 9b, c. They are most prominent in the dorsal arms where, in the distal half of the arm, they range in diameter from about 0.2–0.6 mm. The width of the arms in this section varies from 1.6 to 2.9 mm. In the non-dorsal arms, the axial nerve size range is 0.3–0.7 mm and the arm width varies between 1.4 and 2.4 mm. It should be noted that the compression of the soft tissue prior to fossilization has likely altered the preserved diameter of these elements.

The suckers of *V. infernalis* are radially symmetrical, and each has a conical, *Vampyroteuthis*-like attachment. There is no clear attachment to the internal arm musculature.

*Plesioteuthis prisca* (Rüppell, 1829).

(Figs. 10, 11, 12)

**Fig. 10:**
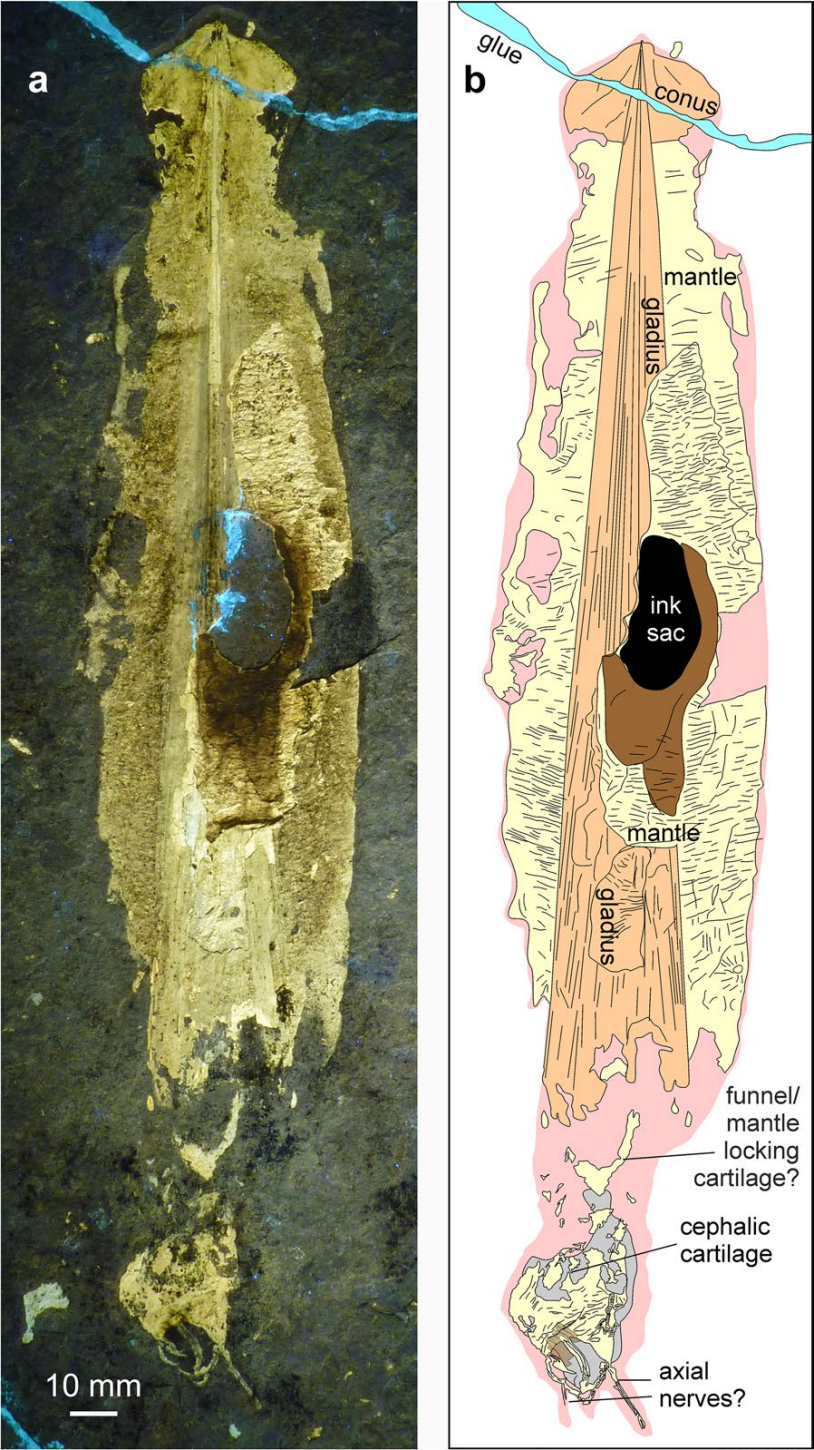
*Plesioteuthis prisca* (Rüppell, 1829), HT 77/23, Daiting (Germany), Tithonian Moernsheim Formation. Specimen is 317 mm long

**Fig. 11:**
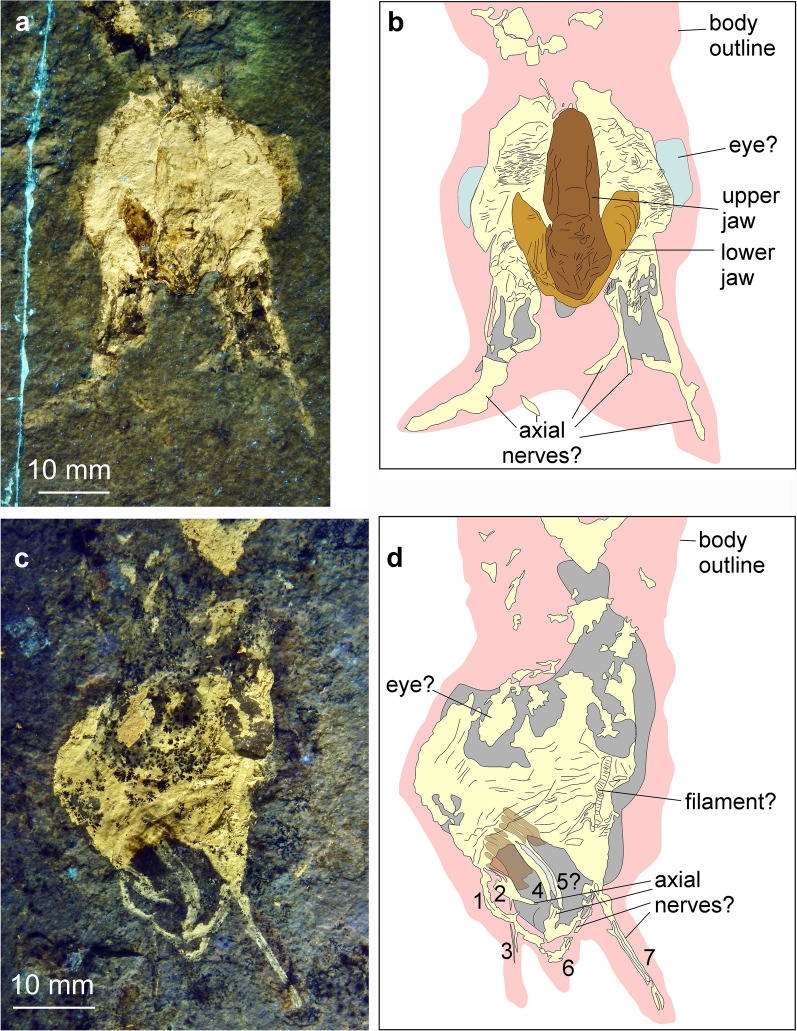
*Plesioteuthis prisca* (Rüppell, 1829), Daiting (Germany), Tithonian Moernsheim Formation. **a**, **b** HT 73/152 **c**, **d** HT 77/23 detail of Fig. 8

**Fig. 12:**
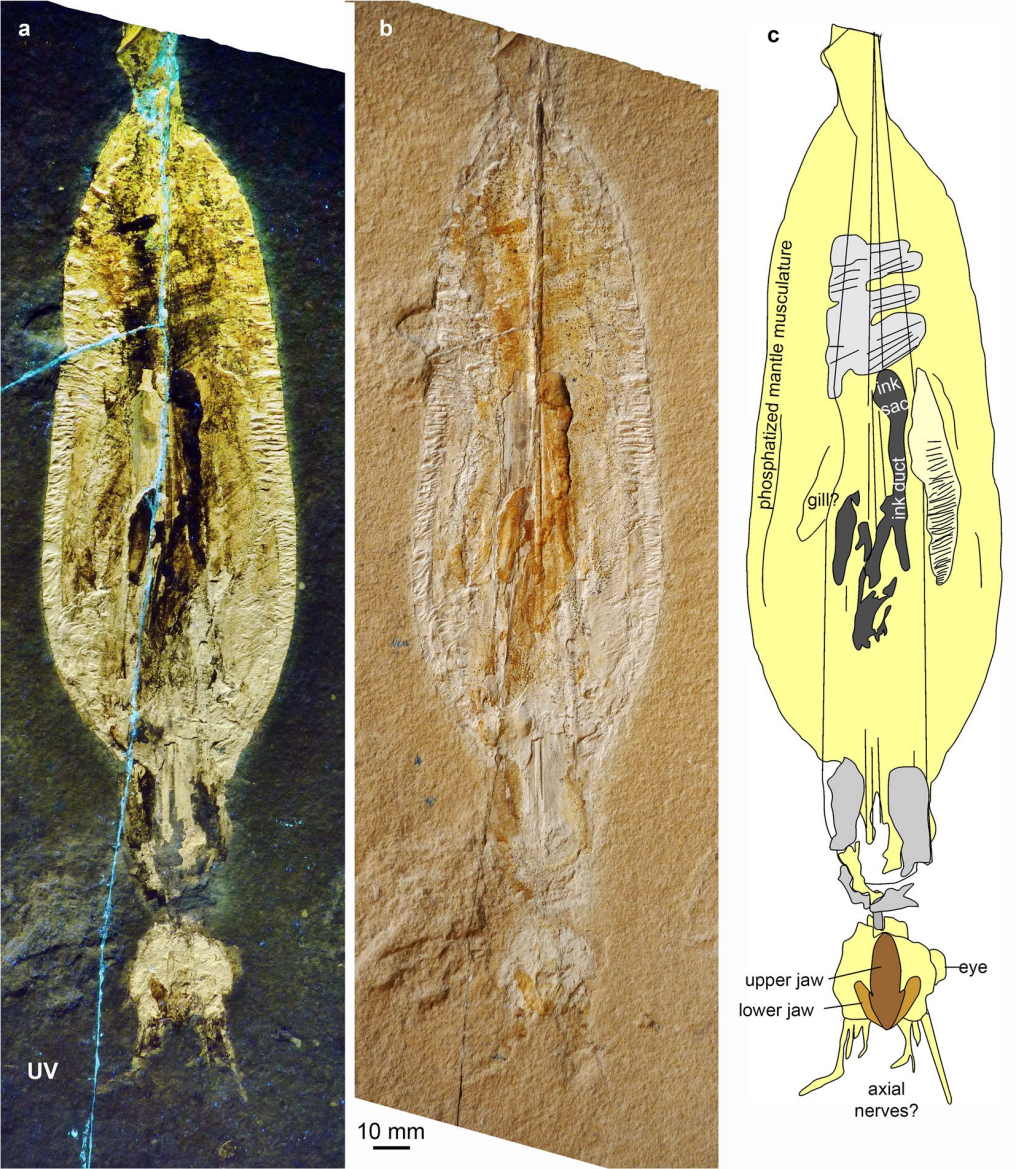
*Plesioteuthis prisca* (Rüppell, 1829), HT 73/152, Daiting (Germany), Tithonian Moernsheim Formation. **a** UV-light. **b** White light. **c** Interpretative sketch

*Specimens* HT 73/152, HT 77/23 (col. H. Tischlinger) .

*Stratigraphy* Moernsheim Formation, Moernsheimensis subzone, *Hybonotum* Zone, Lower Tithonian, Jurassic.

*Locality* Daiting near Monheim, Bavaria, Germany.

*Description of arm crown* Two specimens of *P. prisca* are discussed here because they both display a peculiarly preserved arm crown. HT 77/23 (Figs. 10, 11c, d) is a complete specimen measuring 317 mm from arm tip to the tip of the gladius. The mantle length is approximately 250 mm and partially covers the gladius. The conus is heavily phosphatized like the muscular mantle. The gladius is hardly phosphatized at its anterior edge and hence not well preserved there. The ink sac and duct are clearly visible. The head (Fig. 11c, d) is preserved on a slight angle, possibly due to necrolytical processes. It displays an oval structure, 13 mm long and 11 mm wide, which we interpret to be an imprint of the cephalic cartilage. The arm crown is preserved as seven phosphatized elongate structures of about 1 mm width each. All but one arm are curled inward, as seen in an exceptionally preserved specimen from the Kimmeridgian of Painten (BMMS 617a, Klug et al., 2015: fig. 2). The Painten-specimen, however, has much thicker arms, which display their cirri. In most other specimens, including those with landing marks (Klug et al., 2015: fig. 7) that sometimes accurately reflect arm proportions, the arms are much thicker proportionally (Additional File 1).

Specimen HT 73/152 is quite similarly preserved (Figs. 11a, b 12). It measures 320 mm from arm tip to gladius tip and also displays a strongly phosphatized mantle, the remains of an ink sac and duct, as well as the gladius. The head displays a limonitic stain on the phosphatic mass, which is here interpreted as jaw remains. Like in HT 77/23, this specimen preserves seven to eight fine, lightly phosphatized structures, which are about 1 mm wide. Correspondingly, these proportions support the interpretation that it is not the arms but rather the axial nerve cords, which are preserved here.

### Ancestral state reconstructions

**Fig. 13:**
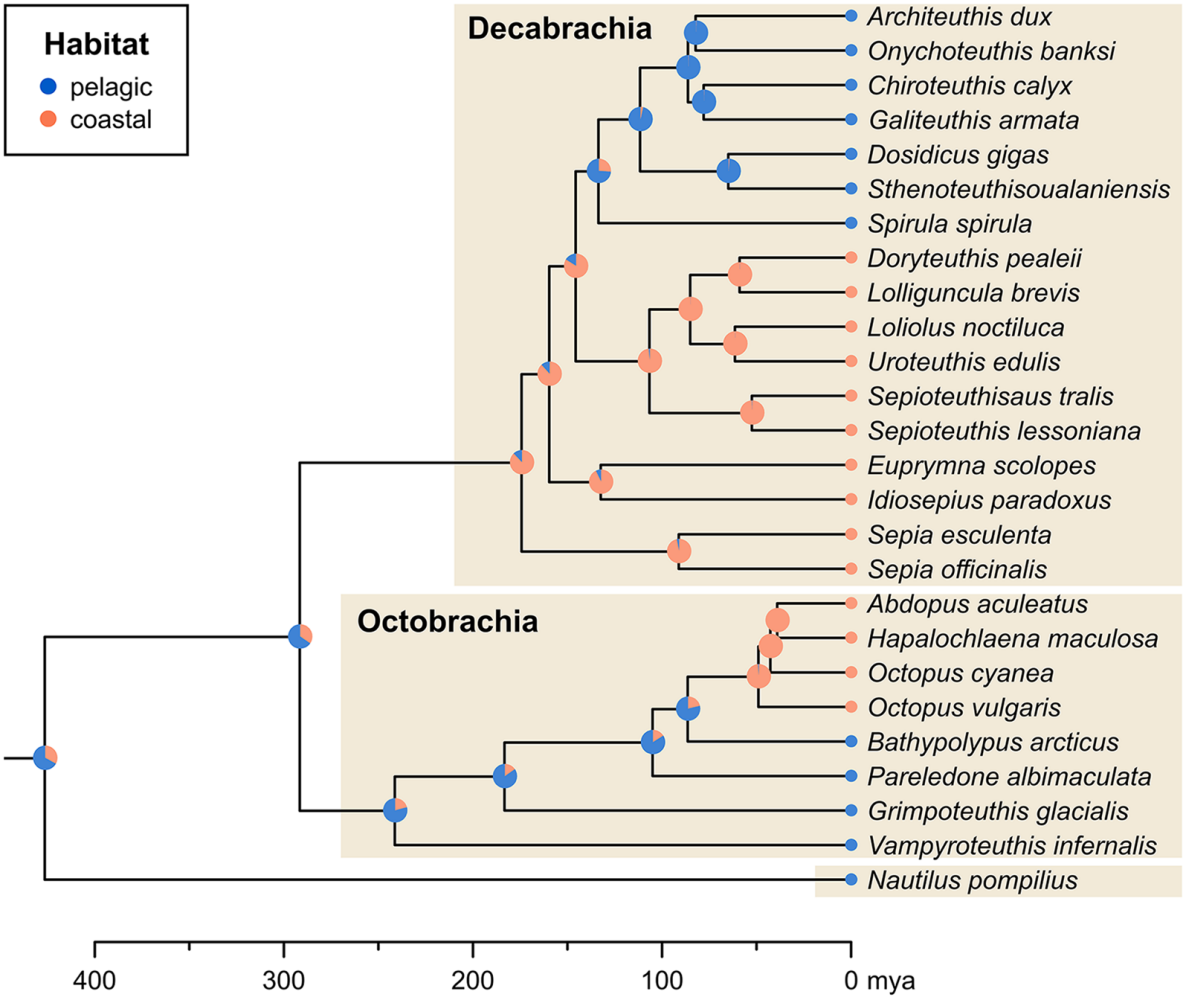
Bayesian ancestral state reconstruction of cephalopod habitat distribution. Tree from Tanner et al., (2017) based on transcriptomic data. Pie charts at nodes represent posterior probabilities of states. Coastal habitats are essentially restricted to the photic zone, while pelagic species are either vertical migrants or deep-sea inhabitants. Note the independent secondary invasion of the pelagic habitat within decabrachians and the shift from pelagic to coastal habitat within octobrachians

The analyses show a strong phylogenetic signal in the habitat distributions of crown group coleoids (Fig. 13). The oldest nodes (crown Cephalopoda and crown Coleoidea) contain the highest uncertainty, both slightly favouring a pelagic habitat with a probability of about 2/3. This high uncertainty likely stems from the necessarily poor sampling of the *Nautilus* lineage, but also from the reconstruction of the basal coleoid dichotomy, where the crown octobrachian node is reconstructed with high probability (79%) as pelagic, while the most recent common ancestor of crown decabrachians most likely (88%) had a coastal habitat. Within both superorders, our analysis recovered a single habitat transition. Within Octobrachia, this switch occurred at the base of the Octopodidae, which is reconstructed as coastal with high probability (78%). Conversely, the transition between coastal and pelagic decabrachians was estimated at the node containing Oegopsida + Spirulida (74%). The transition rate for the change in habitat was estimated to a mean of 0.0019 per million years, with a median of 0.0017 and a 95% highest posterior density interval between 0.0002 and 0.0043. Thus, on average, a single lineage would be expected to transition between habitats only once in 500 million years, indicating a very slow transition rate.

## Discussion

### Taphonomy

In the past decades, experimental studies such as those of Clements et al., (2017) have shown the differential decay and preservation potential of coleoid organs. Clements et al., (2017: fig. 4) found that the mantle was quite resistant to decay, which coincides with the fact that it is often preserved in platy limestone Lagerstätten such as Solnhofen-Eichstätt (Germany), Hadjoula and Haqel (Lebanon), etc. They observed a significant difference in the rate of decay in the arms of octobrachians versus decabrachians. This was reflected in the pH levels, which stayed conducive to phosphatization in octobrachians, though was too high for preservation in the decabrachians (Clements et al., 2017: fig. 2). Interestingly, suckers of decabrachians were lost after three days, while octobrachians appear to be taphonomically more resilient. Despite the differences in necrolysis and the according character loss, a fast burial is still required to obtain the superior preservation seen in the coleoids from La Voulte-sur-Rhône (Fuchs & Hoffmann, 2017; Kruta et al., 2016; Rowe et al., 2022, 2023). The decabrachian tissue connecting the head and mantle decayed after about one week, and the mantle tissue supporting the anterior margin of the gladius broke down after about 10 days. These results are consistent with the states observed in the two *Plesioteuthis prisca* specimens depicted in Figs. 11 and 12 where the head in HT 77/23 is offset from the body, and the gladius extends anteriorly beyond the mantle in both specimens.

The selective preservation of axial nerves seen here is supported by the results of decay experiments on chordates by Sansom et al., (2010). They observed that the dorsal nerve chord, (i.e. nerve tissue), was preserved for 130 days. Accordingly, the preferential preservation of the principal arm nerves when compared with mantle tissue becomes understandable. The correctness of their interpretation is, to some degree, supported by the reasonably widespread preservation of the cephalopod cephalic cartilage in Mesozoic coleoids and maybe even in some exceptionally preserved ammonoids (e.g., Fuchs, 2006a, 2006b; Fuchs & Larson, 2011a, 2011b; Klug et al., 2012, 2016, 2019; Jattiot et al., 2015; Klug & Lehmann, 2015; Donovan & Fuchs, 2016; Lukeneder & Lukeneder, 2022).

### Homologization of body parts

Identification and homologization of fossilized soft parts is often challenging. This is due, in part, to the selective preservation of tissues, the different modes of preservation (including mineralization), and compression prior to fossilization, which is quite common in conservation deposits such as black shales and platy limestones. Hence, special attention must be paid to homologization.

The axial nerve cords can be identified based on their position, both the location within the arm crown and their association with the rows of arm hooks, as well as their structure, though this is more difficult to assess as nerve cells are not preserved and there are no surface structures to support the interpretation. Nevertheless, in these specimens, the number, diameter in relation to arm thickness, and the association with the head region suggest that both criteria are fulfilled. In the vampyromorphs from the Middle Jurassic of La Voulte-sur-Rhône, the axial nerve cords are actually preserved in three dimensions and still are surrounded by the arm musculature, thus confirming their nature (Kruta et al., 2016; Rowe et al., 2022, 2023). The criteria of embryology and continuity can only partially be evaluated. The embryonic development of the discussed fossil coleoids is unknown. Nevertheless, the presence of neuronal strands in the arms is well-documented in recent coleoid embryos (Shigeno et al., 2001: fig. 2). By contrast, continuity is given since the axial nerve cords are now documented from several important clades from the fossil record (Fig. 13).

### Evolution of the arm crown and its nervous system

The oldest arm crowns of coleoids are those of *Gordoniconus beargulchensis* (AMNH 50267/AMNH 43264) from the Carboniferous of Montana (Klug et al., 2019 and references therein; Whalen & Landman, 2022). The early coleoid fossil *Syllipsimopodi bideni*, published by Whalen and Landman (2022: fig. 4), shares size, proportions, conch shape, preservation, locality and stratum with the conspecific *G. beargulchensis* as supposed by Klug et al. (in press). Their specimen (ROMIP 64897) preserves an arm crown, corroborating the finding of Klug et al. (2019) that this species had ten arms. It appears that the arm length was not uniform in this species (primarily or as a taphonomic artefact?), similar to the Middle Jurassic vampyromorphs from La Voulte-sur-Rhône (Kruta et al., 2016; Rowe et al., 2022). All arms were described with small suckers.

To date, no traces of the nervous system in fossilized arm crowns are known from the Palaeozoic (arm hooks: Doguzhaeva et al., 2007). Although phragmoteuthid arm crowns are reasonably well documented from the Triassic (Doguzhaeva et al., 2018; Fuchs & Donovan, 2018; Fuchs, 2006a; Lukeneder & Lukeneder, 2022; Rieber, 1970), they do not show the axial nerve cords. The oldest axial nerves described in the literature belong to the Toarcian *Chondroteuthis wunnenbergi* presented here.

The Middle Jurassic vampyromorphs from La Voulte-sur-Rhône (Kruta et al., 2016; Rowe et al., 2022, 2023) represent the most convincing examples of preserved axial nerves cords and provide the greatest anatomical detail. Although the intrabrachial ganglia are indiscernible in the fossil material, the morphology of the suckers in these specimens is now well documented; the large suckers with a large infundibulum might indicate the presence of ganglia in front of each sucker as in modern octobrachians. This suggests that these Jurassic forms already had the ability to coordinate precise movements. In contrast, nautilids lack the brain structure for an elaborate control of the arm (Budelmann, 1995). The digital tentacles and cirri of modern nautilids have an axial nerve cord (Kier, 2010; Nixon & Young, 2003) but lack suckers and the associated intrabrachial ganglia. Noteworthy, upon bait stimulation the grooved tentacle tips show a first initial chemosensory response followed by a tactile one (Nixon & Young, 2003). The type of arm armature and how the arms are used could therefore be related to the complexity and structure of the central nervous system. *Nautilus* is considered to have a much simpler nervous system compared with other cephalopods, while octobrachians have larger brachial and pedal lobes compared to decabrachians due to their advanced use of arms (Budelmann, 1995). Although fossil central nervous systems are rare, a complex arm armature could therefore provide indirect evidence for the complexity of the nervous system. The data on arm armature (suckers, hooks) and remnants of the axial nerve cords seem to indicate complex nervous systems were already present in the Palaeozoic.

Studies of the evolution of the nervous system in Palaeozoic cephalopods are hampered by the fact that even if arm crowns were discovered, the placement of the origin of the *Nautilus* lineage is uncertain (compare, e.g., Kröger et al., 2011; Pohle et al., 2022). This complicates interpretations of evolutionary transitions near the cephalopod crown group.

### Ecological implications

Cephalopods are world-renowned for comprising some of the most intelligent invertebrates (e.g., Budelmann, 1995; Crook & Basil, 2008; Nixon & Young, 2003; Schnell et al., 2021). What ecological drivers brought molluscs to this point? We cannot provide a satisfying reply to that question yet. However, we hypothesize that a combination of factors was at play, including changes in locomotion, habitat, and the need to process sensory input from an increased number of sensory cells, as well as ecological factors such as rising predatory pressure (e.g., Klug et al., 2017; Vermeij, 1977). The data on the axial nerve cords, which we present here, suggest that the nervous system was already highly evolved in early coleoids (see also Klug et al., 2019) far back in the Palaeozoic.

**Fig. 14:**
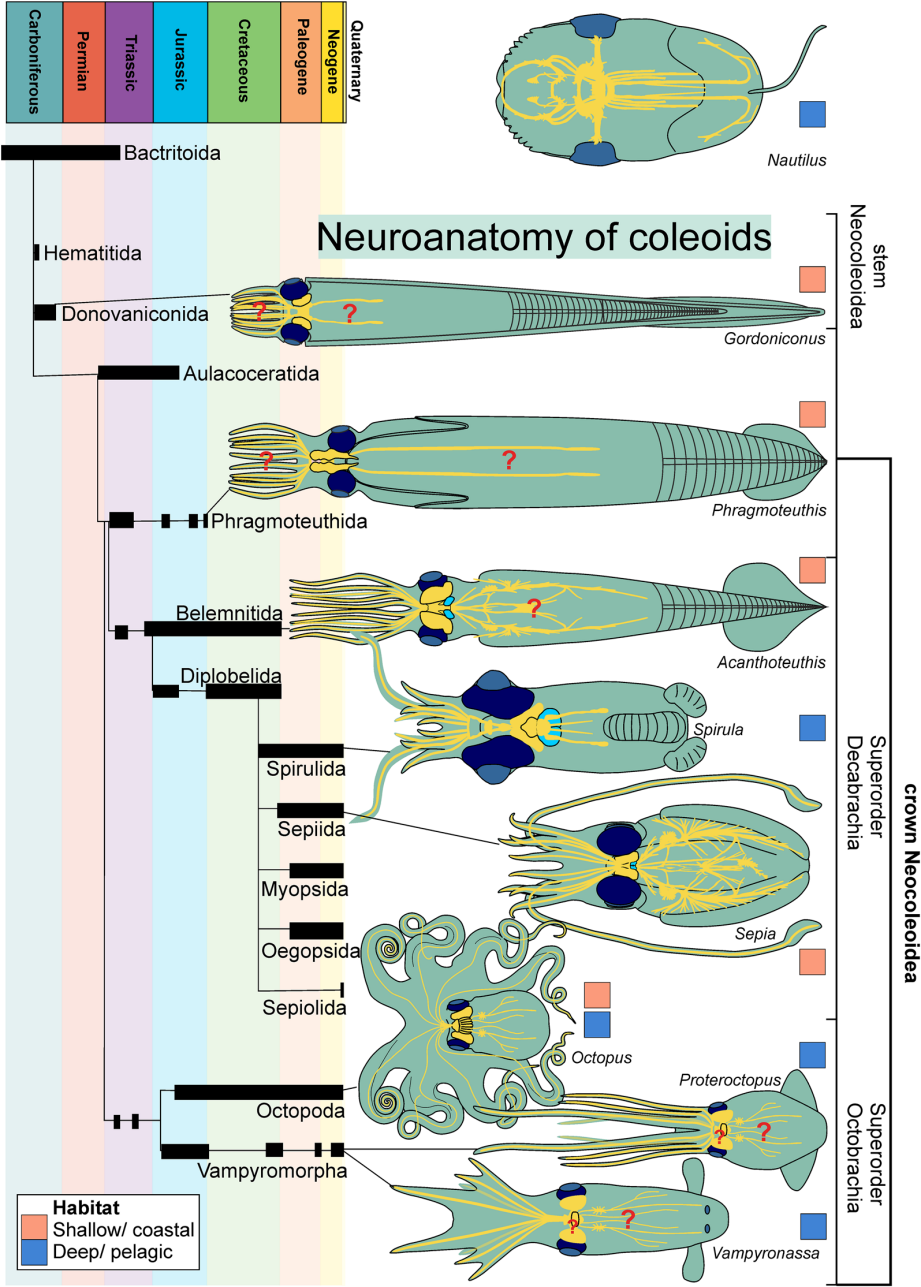
Coleoid phylogeny (modified after Kröger et al., 2011) with illustrations of the nervous system (yellow), eyes (blue) and statocysts (turquoise) of some fossil and modern representatives of important coleoid clades. *Nautilus* after Budelmann (1996: fig. 4), *Gordoniconus* after Klug et al., (2019), *Phragmoteuthis* after Lukeneder and Lukeneder, (2022), *Acanthoteuthis* after material presented here and Klug et al., (2016), *Spirula* after Huxley and Pelseneer, (1895) and Trombke, (2016: fig. 2, 7–9), *Sepia* after Budelmann, (1996: fig. 1), *Octopus* after Haeckel (1904: pl. 54) and Chung et al., (2022a, 2022b: fig. 6, 7), *Proteroctopus* after Kruta et al., (2016), *Vampyronassa* after Rowe et al., (2022: fig. 1, 5)

The axial nerve cords did not deliver much information regarding likely ecological drivers of coleoid evolution. Unsurprisingly, the brain has a larger potential there. As demonstrated by Nixon and Young, (2003), the brains of cephalopods are as diversified as the group itself. In Fig. 14, we present a first overview of the nervous systems of cephalopods including axial nerves and the brains with the optical lobes through phylogeny. So far, not much is known about the nervous system in the body, hence these were tentatively reconstructed based on close relatives. Eye-size varies quite substantially in relation to body size and of the depicted taxa: the deepest diver, *Spirula spirula*, has the largest eyes (e.g., Huxley & Pelseneer, 1895; Nixon & Young, 2003; Trombke, 2016). Surprisingly, the optic lobes vary much less in size than the eyes, although the brain of *Spirula* also appears large in relation to body size. When only the body chamber or soft body in the proostracum region is considered in phragmocone-bearing forms, they also have rather large brains in relation to body (chamber) size.

Remarkably, *Spirula* appears to have rather large statocysts when compared with other cephalopods (Huxley & Pelseneer, 1895; Trombke, 2016). This seems logical given they inhabit the deeper parts of the water column where orientation is hampered by low light conditions and the distance to the sea-floor. By contrast, one might hypothesize that phragmocone-bearing forms are permanently informed about their spatial orientation by their gas-filled phragmocone and thus orient themselves with the gas-filled space usually situated above (e.g., Denton & Gilpin-Brown, 1966; Hoffmann et al., 2015; Jacobs, 1996; Naglik et al., 2016; Peterman et al., 2021; Tajika et al., 2015; Ward, 1979, 1987; Ward & Martin, 1978). Of course, the statocysts also allow the animal to orient itself in the water, both for swimming direction and vertical movement. Hence, sensitive statocysts are more important for non-benthic forms that are nocturnal and/or migrate below the euphotic zone. In the case of *Spirula*, it was recently demonstrated that they occasionally swim with their head facing upward (Lindsay et al., 2020), which seems counter-intuitive since balancing on the gas-filled phragmocone must be difficult and an inefficient use of energy. Nevertheless, the spirulid phragmocone contains a considerable amount of chamber liquid, thereby facilitating these movements. Of course, a refined sense of spatial orientation helps minimize the energetic cost of such actions.

Chung et al., (2022a, 2022b) discovered relationships between brain structure in modern cephalopods and habitat as well as habit. According to these authors, octobrachians with subdivided optic lobes and 7-gyrus vertical lobes are characteristically diurnal species that inhabit the photic zone, while nocturnal species have bean-shaped optic lobes and 5-gyrus vertical lobes (Chung et al., 2022a). Similarly, croissant-shaped optic lobes are typical for diurnal sepiids, while they are bean-shaped in nocturnal species (Chung et al., 2022b). *Vampyroteuthis* has a simple vertical lobe and bean-shaped optic lobes and is a deep-sea dweller. This suggests that the bean-shaped optic lobes are typical for forms that live under low light conditions.

Transferring this knowledge of Chung et al., (2022a, 2022b) to extinct coleoids is hampered by the usually insufficient preservation of the neuroanatomy and the impossibility to observe their behaviour. Additionally, while the host rock of fossils informs us to some degree about the palaeoenvironment but it does not allow conclusions on the exact habitat depth especially when the species under consideration was not benthic or demersal. Our ancestral state reconstructions (Fig. 13) of habitat suggest that light conditions played an important role in coleoid evolution, as switches between shallow and at least partly deep habitats occurred relatively rarely. We are aware that the ancestral state reconstructions have several shortcomings such as the relatively low species coverage, reliance on a single tree despite considerable uncertainties in coleoid phylogenetics (see, e.g., Tanner et al., 2017; Sanchez et al., 2018; Anderson & Lindgren, 2021; Lindgren et al., 2022) and the use of a relatively simplistic model that assumes equal transition rates. Despite this, the signal for the transitions between coastal and pelagic species is quite strong. Therefore, we suggest that these factors do not greatly impact the overall result, though they might contribute a higher amount of uncertainty.

Although we did not include fossil taxa in the ancestral state reconstructions (Fig. 13), we can obtain some information on their habitats from the sediment and associated fauna. This can then be compared to their assumed phylogenetic positions as an independent test.

Concerning the oldest complete coleoid *Gordoniconus* (Klug et al., 2019), the associated fauna suggests a photic zone habitat (Horner & Hanson, 2020). Eyes are not preserved but fragments of the cephalic cartilage are. With some reservation, we suggest a pelagic and nocturnal mode of life, because we assume that their bactritid and orthocerid ancestors had a rather passive nektoplanktic mode of life as vertical migrants (Kröger, 2003), which rose to shallower water during the night. A potential bias roots in drifting conchs (e.g., Wani et al., 2005; Yacobucci, 2018). However, *Gordoniconus* is fairly common, and several specimens preserve soft tissue-remains (Klug et al., 2019), which are unlikely to be preserved in drifted shells.

Phragmoteuthids are known from several Triassic localities in the Alps such as Polzberg (Lukeneder & Lukeneder, 2021, 2023). Fauna, flora and facies suggest an epipelagic to mesopelagic environment. Their widely accepted phylogenetic position as the last common ancestors of Decabrachia and Octobrachia coincides with the possibly predominantly coastal habitat of stem decabrachians recovered in our Bayesian ancestral state reconstruction of cephalopod habitat distribution (Fig. 13). The cephalic cartilage is unusually well preserved (Lukeneder & Lukeneder, 2022) and displays a quite constricted shape of the optic lobe, providing the anatomical interpretation of these strange cartilages are correct. Chung et al., (2022a, 2022b) found that diurnal octobrachians have distinctly constricted optic lobes while in nocturnal taxa, the optic lobes are more bean-shaped. Presuming the interpretation of its anatomy is correct, *Phragmoteuthis* would thus have been diurnal.

The cephalic cartilage of the stem decabrachiate *Acanthoteuthis* is reasonably well known (Klug et al., 2016: fig. 1b). It is quite constricted, suggesting a diurnal mode of life in the lagoons of the Solnhofen-Eichstätt-Nusplingen archipelago. The same likely applies to the Cretaceous stem octobrachiate *Dorateuthis syriaca* from Lebanon (Fuchs & Larson, 2011a, 2011b). The question arises whether diurnal vertical migration is likely in basins with oxygen impoverished bottom waters. This might be explained by the fact that some organisms do not sink below the twilight zone (Watanabe et al., 2006; Häfker et al., 2017; Kaartvedt et al., 2020), but of course, the lagoons of the Solnhofen archipelago were likely less deep (Kölbl-Ebert & Cooper, 2019). In any case, moving to deeper and thus darker parts of the sea during daylight still was an important strategy to escape predation. The more coastal habitat of many living decabrachians groups coincides with the coastal habitat of stem decabrachians of Germany and Lebanon.

In several respects, the vampyromorphs from the Middle Jurassic of La Voulte-sur-Rhône show the best soft tissue preservation currently known (Kruta et al., 2016; Rowe et al., 2022, 2023). However, their cephalic cartilage is hardly discernible in the tomography images. Despite this, the complex palaeoenvironment of La Voulte-sur-Rhône “*represents a bathyal ecosystem in an offshore environment with steep, fault-controlled bathymetric gradients*” (Rowe et al., 2022: p. 1; see also: Charbonnier, 2009; Charbonnier et al., 2014). Although the fossil assemblage has yielded rare arthropods that are adapted to (eu) photic conditions (Vannier et al., 2016), it is likely that the cephalopod species considered in the present study had a rather deep and certainly pelagic habitat. The general anatomical similarity of at least *Proteroctopus* and *Vampyronassa* to the modern *Vampyroteuthis* suggests a life below the photic zone although we lack direct evidence for the habitat depth (Charbonnier et al., 2014). The supposed photophores of *Vampyrofugiens atramentum* (Rowe et al., 2023) at least suggest a partially dark habitat like in modern *Vampyroteuthis*. Palaeobathymetry often suffers from poor evidence and in the case of the La Voulte-sur-Rhône Lagerstätte, the vampyromorphs have been used as evidence for a bathyal habitat. The different facies and palaeogeographic setting of the La Voulte-sur-Rhône and Solnhofen regions reflect the contrasting depositional depths of the two localities: the complex bathymetry and mix of habitats at La Voulte-sur-Rhône, and the shallow platy limestones of the Solnhofen region were laid down in shallower water. In turn, this suggests a certain diversity in habitat depths of extinct vampyromorphs (e.g., Košťák et al., 2021; Klug et al., 2021b), which is not surprising taking the higher diversity of Jurassic vampyromorphs (about four families with ten genera; see Fuchs et al., 2020) as opposed to the single living species into account.

Overall, neuroanatomical data of Palaeozoic and Mesozoic coleoids are still very patchy (Fig. 14). Based on this sparse evidence and the ancestral state reconstructions, we suggest the following hypotheses, which require more anatomical data of additional species to be adequately tested:

Hypothesis 1: The earliest coleoids (Hematitida, Donovaniconida,? Aulacoceratida) had a tube-like body chamber, which lacked a long forward projecting proostracum. Their bactritid ancestors were diurnal vertical migrants, possibly spending the days in the deep and the nights in shallower water.

Hypothesis 2: Proostracum-bearing predatory ten-armed coleoids (Phragmoteuthida, Belemnitida, Diplobelida) of the Triassic and Jurassic were diurnal and inhabited photic zone habitats.

Hypothesis 3: The neocoleoid crown group originated from diurnal forms of the photic zone.

Hypothesis 4: The split into decabrachians and octobrachians was initially linked with a preference for shallower habitats in decabrachians and for deeper habitats in octobrachians. Both clades diversified ecologically later with subclades living in coastal and pelagic habitats in both coleoid clades.

## Conclusions

We document neuroanatomical details of several Mesozoic coleoid species, which were poorly known previously. For example, we portray the axial nerve cords of *Acanthoteuthis*, *Belemnotheutis*, *Chondroteuthis, Plesioteuthis, Proteroctopus, Sueviteuthis* and *Vampyronassa*. Axial nerve cord preservation varies from 3D in the material from la Voulte and flattened phosphatized lines in the other Jurassic materials. We discuss the preservation modes and taphonomy of the arm crown of Mesozoic coleoids.

The new data are combined with data from the literature to provide an overview of the neuroanatomy of several important extinct and extant coleoid clades. The comparison with modern octobrachians and ancestral state reconstructions suggest that a differentiation in habitat depth and diurnal versus nocturnal mode of life might have played an important role in the evolution of the Coleoidea. We propose four hypotheses concerning these evolutionary processes, which require further coleoid species preserved with neuroanatomical detail to test them.

### Supplementary Information

**Additional file 1.** This folder contains all data used for an produced by the Bayesian ancestral state reconstructions of cephalopod habitat distribution.

## Data Availability

The specimens are stored in the collections of the Geological Institute of the University of Tübingen, Germany (GPIT Ce 1564/2,6/PV-67025), in the collection of Helmut Tischlinger, Stammham, Germany (HT numbers; will be given to a public institution at a later date), in the Etches collection in Kimmeridge, UK (KI306), in the Natural History Museum, London, UK (NHMUK 25966) and in the Bundesanstalt für Geowissenschaften und Rohstoffe (BGR MA 13436). *Proteroctopus* (MNHN.F.R03801) and *Vampyronassa* (MNHN.F.B74244) are deposited in the Muséum national d’Histoire naturelle, Paris. Files for the Bayesian ancestral state reconstruction of cephalopod habitat distribution presented in Fig. 13 are available at XX.

## References

[CR1] Anderson FE, Lindgren AR (2021). Phylogenomic analyses recover a clade of large-bodied decapodiform cephalopods. Molecular Phylogenetics and Evolution.

[CR3] Bode A (1933). *Chondroteuthis wunnenbergi* n. g., n. sp., eine neue Belemnoideenform, in günstiger Erhaltung. Jahresbericht Des Niedersächsischen Geologischen Vereins Zu Hannover.

[CR4] Budelmann BU, Breidbach O, Kutsch W (1995). The cephalopod nervous system: What evolution has made of the molluscan design. The nervous systems of invertebrates: An evolutionary and comparative approach.

[CR5] Budelmann BU (1996). Active marine predators: The sensory world of cephalopods. Marine and Freshwater Behaviour and Physiology.

[CR6] Buresch KC, Sklar K, Chen JY, Madden SR, Mongil AS, Wise GV, FBoal JG, Hanlon RT (2022). Contact chemoreception in multi-modal sensing of prey by Octopus. Journal of Comparative Physiology A.

[CR7] Charbonnier S (2009). Le Lagerstätte de La Voulte: un environnement bathyal au Jurassique.

[CR8] Charbonnier S, Audo D, Caze B, Biot V (2014). The La Voulte-sur-Rhône Lagerstätte (Middle Jurassic, France). Comptes Rendus Palevol.

[CR9] Chase R, Wells MJ (1986). Chemotactic behaviour in *Octopus*. Journal of Comparative Physiology A.

[CR10] Chung W-S, Kurniawan ND, Marshall NJ (2022). Comparative brain structure and visual processing in octopus from different habitats. Current Biology.

[CR11] Chung W-S, López-Galán A, Kurniawan ND, Marshall NJ (2022). The brain structure and the neural network features of the diurnal cuttlefish *Sepia plangon*. iScience.

[CR12] Chung W-S, Marshall NJ (2017). Complex visual adaptations in squid for specific tasks in different environments. Frontiers in Physiology.

[CR13] Clements T, Colleary C, De Baets K, Vinther J (2017). Buoyancy mechanisms limit preservation of coleoid cephalopod soft tissues in Mesozoic Lagerstätten. Palaeontology.

[CR14] Crook R, Basil J (2008). A biphasic memory curve in the chambered nautilus, *Nautilus pompilius* L. (Cephalopoda: Nautiloidea). Journal of Experimental Biology.

[CR15] De Baets K, Klug C, Korn D, Bartels C, Poschmann M (2013). Emsian Ammonoidea and the age of the Hunsrück Slate (Rhenish Mountains, Western Germany). Palaeontographica A.

[CR16] Denton EJ, Gilpin-Brown JB (1966). On the buoyancy of the pearly Nautilus. Journal of the Marine Biological Association UK.

[CR17] Doguzhaeva LA, Brayard A, Goudemand N, Krumenacker LJ, Jenks JF, Bylund KG, Fara E, Olivier N, Vennin E, Escarguel G (2018). An Early Triassic gladius associated with soft tissue remains from Idaho, USA—a squid-like coleoid cephalopod at the onset of Mesozoic Era. Acta Palaeontologica Polonica.

[CR18] Doguzhaeva LA, Mapes RH, Mutvei H, Landman NH, Davis RA, Mapes RH (2007). A Late Carboniferous coleoid cephalopod from the Mazon Creek Lagerstätte (USA), with a radula, arm hooks, mantle tissues, and ink. Cephalopods—present and past. New insights and fresh perspectives, 121-143.

[CR19] Donovan DT, Crane MD (1992). The type material of the Jurassic Cephalopod *Belemnotheutis*. Palaeontology.

[CR20] Donovan DT, Fuchs D (2016). Fossilized soft tissues in Coleoidea. Treatise Online Part M Chapter.

[CR21] Dröscher A (2016). Pioneering studies on cephalopod’s eye and vision at the stazione zoologica Anton Dohrn (1883–1977). Frontiers in Physiology.

[CR23] Fischer JC, Riou B (1982). Le plus ancien Octopode connu (Cephalopoda, Dibranchiata): Proteroctopus ribeti nov gen., nov. sp., du Callovien de l’Ardèche (France). Comptes Rendus De L’académie Des Sciences.

[CR24] Fischer J-C, Riou B (2002). *Vampyronassa rhodanica* nov. gen. nov sp., vampyromorphe (Cephalopoda, Coleoidea) du Callovien inférieur de La Voulte-sur-Rhône (Ardèche, France). Annales De Paléontologie.

[CR25] Frisch K (1912). Über Färbung und Farbensinn der Tiere. Sitzungs-Berichte Gesellschaft Für Morphologie Und Physiologie München.

[CR26] Fuchs D (2006). Fossil erhaltungsfähige Merkmalskomplexe der Coleoidea (Cephalopoda) und ihre phylogenetische Bedeutung. Berliner Paläobiologische Abhandlungen.

[CR27] Fuchs D (2006). Morphology, taxonomy and diversity of vampyropod coleoids (Cephalopoda) from the upper cretaceous of Lebanon. Memorie Della Società Italiana Die Scienze Naturali.

[CR28] Fuchs D (2016). The gladius and gladius vestige in fossil Coleoidea. Treatise Online Pt M Chapter.

[CR29] Fuchs D, Boletzky SV, Tischlinger H (2010). New evidence of functional suckers in belemnoid coleoids weakens support for the “Neocoleoidea” concept. Journal of Molluscan Studies.

[CR30] Fuchs D, Donovan DT (2018). Systematic descriptions: Phragmoteuthida. Treatise Online Pt M Chapter.

[CR31] Fuchs D, Hoffmann R (2017). Arm armature in belemnoid coleoids. Treatise Online Pt M Chapter.

[CR300] Fuchs, D., Laptikhovsky, V., Nikolaeva, S., Ippolitov, A., & Rogov, M. 2020. Evolution of reproductive strategies in coleoid mollusks. *Paleobiology*, *46*, 82–103. 10.1017/pab.2019.41

[CR32] Fuchs D, Hoffmann R, Klug C (2021). Evolutionary development of the cephalopod arm armature: A review. Swiss Journal of Palaeontology.

[CR33] Fuchs D, Larson NL (2011). Diversity, morphology, and phylogeny of coleoid cephalopods from the upper Cretaceous Plattenkalks of Lebanon—Part I: Prototeuthidina. Journal of Paleontology.

[CR34] Fuchs D, Larson NL (2011). Diversity, morphology and phylogeny of coleoid cephalopods from the upper Cretaceous Plattenkalks of Lebanon—Part II: Teudopseina. Journal of Paleontology.

[CR35] Graziadei P (1962). Receptors in the suckers of *Octopus*. Nature.

[CR36] Haas W (1997). Der Ablauf der Entwicklugsgeschichte der Decabrachia (Cephalopoda, Coleoidea). Palaeontographica A.

[CR37] Haeckel E (1866). Generelle Morphologie der Organismen. Allgemeine Grundzüge der organischen Formen-wissenschaft, mechanisch begründet durch die von Charles Darwin reformirte Descendenztheorie.

[CR38] Haeckel E (1904). Kunstformen der Natur.

[CR39] Häfker NS, Meyer B, Last KS, Pond DW, Hüppe L, Teschke M (2017). Circadian clock involvement in zooplankton diel vertical migration. Current Biology.

[CR40] Hess C (1902). Über das Vorkommen von Sehpurpur bei Cephalopoden. Centralblatt Physiologie.

[CR41] Hess C (1905). Beiträge zur Physiologie und Anatomie des Cephalopodenauges. Pflügers Archiv Der Gesellschaft Für Physiologie.

[CR42] Hess C (1912). Vergleichende Physiologie des Gesichtssinnes.

[CR43] Hoffmann R, Howarth MK, Fuchs D, Klug C, Korn D (2022). The higher taxonomic nomenclature of Devonian to Cretaceous ammonoids and Jurassic to Cretaceous ammonites including their authorship and publication. Neues Jahrbuch Für Geologie Und Paläontologie, Abhandlungen.

[CR44] Hoffmann R, Lemanis R, Naglik C, Klug C, Klug C, Korn D, De Baets K, Kruta I, Mapes RH (2015). Ammonoid buoyancy. Ammonoid paleobiology, Volume I: from anatomy to ecology.

[CR47] Hoffmann R, Weinkauf MFG, Fuchs D (2017). Grasping the shape of belemnoid arm hooks—a quantitative approach. Paleobiology.

[CR48] Höhna S, Landis MJ, Heath TA, Boussau B, Lartillot N, Moore BR, Huelsenbeck JP, Ronquist F (2016). RevBayes: Bayesian phylogenetic inference using graphical models and an interactive model-specification language. Systematic Biology.

[CR49] Horner JR, Hanson DA (2020). Vertebrate paleontology of Montana. MBMG Special Publication. Geology of Montana.

[CR50] Huxley, T. H., & Pelseneer, P. (1895). *Zoölogy of the Voyage of H. M. S. Challenger: Part I., XXXII. Report on* Spirula*. VIII*., 32 and 12, pp. 4, 6 pls

[CR51] Jacobs DK (1996). Chambered cephalopod shells, buoyancy, structure, and decoupling: History and red herrings. Palaios.

[CR52] Jattiot R, Brayard A, Fara E, Charbonnier S (2015). Gladius-bearing coleoids from the upper Cretaceous Lebanese Lagerstätten: Diversity, morphology, and phylogenetic implications. Journal of Paleontology.

[CR53] Jeletzky, J. A. (1966). Comparative morphology, phylogeny, and classification of fossil Coleoidea. *University of Kansas Paleontological Contributions, Mollusca, 7*, 162

[CR54] Jeletzky JA (1965). Taxonomy and phylogeny of fossil Coleoidea (=Dibranchiata). Geological Survey of Canada Papers.

[CR55] Kaartvedt S, Røstad A, Christiansen S, Klevjer TA (2020). Diel vertical migration and individual behavior of nekton beyond the ocean’s twilight zone. Deep-Sea Research I.

[CR56] Katz I, Shomrat T, Nesher N (2021). Feel the light: sight-independent negative phototactic response in octopus arms. Journal of Experimental Biology.

[CR58] Kier WM, Saunders WB, Landman NH (2010). The functional morphology of the tentacle musculature of *Nautilus pompilius*. Nautilus: The biology and Paleobiology of a living fossil, reprint with additions.

[CR59] Kingston ACN, Kuzirian AM, Hanlon RT, Cronin TW (2015). Visual phototransduction components in cephalopod chromatophores suggest dermal photoreception. The Journal of Experimental Biology.

[CR60] Kingston ACN, Wardill TJ, Hanlon RT, Cronin TW (2015). An unexpected diversity of photoreceptor classes in the longfin squid Doryteuthis pealeii. PLoS ONE.

[CR61] Klinghardt F (1932). Über den methodischen Nachweis der Eingeweide bei fossilen Tintenfischen. Paläontologische Zeitschrift.

[CR62] Klug C, Davesne D, Fuchs D, Argyriou T (2020). First record of non-mineralized cephalopod jaws and arm hooks from the latest Cretaceous of Eurytania Greece. Swiss Journal of Palaeontology.

[CR63] Klug C, Frey L, Pohle A, De Baets K, Korn D (2017). Palaeozoic evolution of animal mouthparts. Bulletin of Geosciences.

[CR64] Klug C, Fuchs D, Schweigert G, Röper M, Tischlinger H (2015). New anatomical information on arms and fins from exceptionally preserved *Plesioteuthis* (Coleoidea) from the Late Jurassic of Germany. Swiss Journal of Palaeontology.

[CR65] Klug C, Landman NH, Fuchs D, Mapes RH, Pohle A, Gueriau P, Reguer S, Hoffmann R (2019). Anatomy of the first Coleoidea and character evolution in the Carboniferous. Communications Biology.

[CR66] Klug C, Lehmann J, Klug C, Korn D, De Baets KI, Mapes RH (2015). Soft part anatomy of ammonoids: reconstructing the animal based on exceptionally preserved specimens and actualistic comparisons. Ammonoid paleobiology, Volume I: from anatomy to ecology Topics in Geobiology.

[CR67] Klug C, Pohle A, Roth R, Hoffmann R, Wani R, Tajika A (2021). Preservation of nautilid soft parts inside and outside the conch interpreted as central nervous system, eyes, and renal concrements from the Lebanese Cenomanian. Swiss Journal of Palaeontology.

[CR68] Klug C, Riegraf W, Lehmann J (2012). Soft-part preservation in heteromorph ammonites from the Cenomanian-Turonian boundary event (OAE 2) in the Teutoburger Wald (Germany). Palaeontology.

[CR69] Klug C, Schweigert G, Fuchs D, De Baets K (2021). Distraction sinking and fossilized Coleoid predatory behaviour from the German Early Jurassic. Swiss Journal of Palaeontology.

[CR70] Klug C, Schweigert G, Fuchs D, Kruta I, Tischlinger H (2016). Adaptations to squid-style high-speed swimming in Jurassic belemnitids. Biology Letters.

[CR71] Klug C, Schweigert G, Tischlinger H, Pochmann H (2021). Failed prey or peculiar necrolysis? Isolated ammonite soft body from the Late Jurassic of Solnhofen (Germany). Swiss Journal of Palaeontology.

[CR72] Klug, C., Stevens, K., Hoffmann, R., Zaton, M., Košťák, M., Weis, R., De Baets, K., Lehmann, J., Fuchs, D., & Vinther, J. Revisiting the identification of *Syllipsimopodi bideni* and timing of the decabrachian-octobrachian divergence. *Nature Communications***(in press)**. 10.1038/s41467-023-42842-x PMC10703834 38062003

[CR73] Kölbl-Ebert M, Cooper BJ (2019). Solnhofener Plattenkalk: A heritage stone of international significance from Germany. Geological Society London Special Publications.

[CR74] Kröger B (2003). The size of siphuncle in cephalopod evolution. Senckenbergiana Lethaea.

[CR75] Kröger B, Vinther J, Fuchs D (2011). Cephalopod origin and evolution: A congruent picture emerging from fossils, development and molecules. BioEssays.

[CR76] Kruta I, Rouget I, Charbonnier S, Bardin J, Fernandez V, Germain D, Brayard A, Landman NH (2016). *Proteroctopus ribeti* in coleoid evolution. Palaeontology.

[CR77] Larson NL, Morton RW, Larson PL, Bergmann U, Tanabe K, Shigeta Y, Sasaki T, Hirano H (2010). A new look at fossil cephalopods. Cephalopods—present and past.

[CR78] Lee PG (1992). Chemotaxis by *Octopus maya* Voss et Solis in a Y-maze. Journal of Experimental Marine Biology and Ecology.

[CR79] Lewis PO (2001). A likelihood approach to estimating phylogeny from discrete morphological character data. Systematic Biology.

[CR80] Lindgren AR, Pratt A, Vecchione M, Anderson FE (2022). Finding a home for the ram’s horn squid: Phylogenomic analyses support *Spirula spirula* (Cephalopoda: Decapodiformes) as a close relative of Oegopsida. Organisms Divsersity & Evolution.

[CR81] Lindsay DJ, Hunt JC, McNeil M, Beaman RJ, Vecchione M (2020). The first in situ observation of the ram’s horn squid *Spirula spirula* turns “common knowledge” upside down. Diversity.

[CR82] Linnaeus, C. (1758). *Systema Naturae per regna tria naturae, secundum classes, ordines, genera, species, cum characteribus, differentiis, synonymis, locis.* 10th revised edition, vol. 1: 824 pp. Laurentius Salvius: Holmiae.

[CR83] Lukeneder A, Lukeneder P (2021). The upper Triassic Polzberg palaeobiota from a marine Konservat-Lagerstätte deposited during the Carnian Pluvial Episode in Austria. Scientific Reports.

[CR84] Lukeneder A, Lukeneder P (2023). New data on the marine upper Triassic palaeobiota from the Polzberg Konservat-Lagerstätte in Austria. Swiss Journal of Palaeontology.

[CR85] Lukeneder P, Lukeneder A (2022). Mineralized belemnoid cephalic cartilage from the late Triassic Polzberg Konservat-Lagerstätte (Austria). PLoS ONE.

[CR86] Mantell, G. A. (1854). *The Medals of Creation: Or, First Lessons in Geology and the Study of Organic Remains*. H.G. Bohn, London.

[CR87] Mantell GA (1848). Observations on some belemnites and other fossil remains of Cephalopoda, discovered by Mr. Reginald Neville Mantell, C.E. in the Oxford Clay near Trowbridge, in Wiltshire. Philosophical Transactions of the Royal Society.

[CR88] Maselli V, Al-Soudy A-S, Buglione M, Aria M, Polese G, Di Cosmo A (2020). Sensorial hierarchy in Octopus vulgaris’s food choice: chemical vs. visual. Animals.

[CR89] Messenger JB (1977). Evidence that *Octopus* is colour-blind. Journal of Experimental Biology.

[CR90] Messenger JB, Wilson AP, Hedge A (1973). Some evidence for colour-blindness in *Octopus*. Journal of Experimental Biology.

[CR91] Mironenko AA, Boiko MS, Bannikov AF, Arkhipkin AI, Bizikov VA, Košťák M (2021). First discovery of the soft-body imprint of an Oligocene fossil squid indicates its piscivorous diet. Lethaia.

[CR92] Münster, G. Zu, G. (1839). Decapoda Macroura Abbildung und Beschreibung der fossilen langschwänzigen Krebse in den Kalkschiefern von Bayern. In: Münster, G. Zu, G. (eds). Beiträge zur Petrefacten-Kunde

[CR93] Naef, A. (1922). *Die fossilen Tintenfische. Eine paläozoologische Monographie.* Jena (Fischer). 322 pp.

[CR94] Naglik C, Rikhtegar FN, Klug C (2016). Buoyancy in Palaeozoic ammonoids from empirical 3D-models and their place in a theoretical morphospace. Lethaia.

[CR95] Nixon M, Young JZ (2003). The brains and lives of cephalopods.

[CR96] Owen R (1844). VI. A description of certain Belemnites, preserved, with a great proportion of their soft parts, in the Oxford Clay, at Christian-Malford, Wilts. Transactions of the Royal Society of London.

[CR97] Pagel M, Meade A, Barker D (2004). Bayesian estimation of ancestral character states on phylogenies. Systematic Biology.

[CR98] Pearce JC (1842). On the mouths of ammonites, and on fossil contained in laminated beds of the Oxford Clay, discovered in cutting the Great Western Railway, near Christian Malford in Wiltshire. Proceedings of the Geological Society of London.

[CR99] Pearce JC (1847). On the fossil Cephalopoda constituting the genus *Belemnotheutis*, Pearce. London Geological Journal.

[CR100] Peterman DJ, Ritterbush KA, Ciampaglio CN, Johnson EH, Inoue S, Mikami T, Linn TJ (2021). Buoyancy control in ammonoid cephalopods refined by complex internal shell architecture. Scientific Reports.

[CR101] Pohle A, Kröger B, Warnock RCM, King AH, Evans DH, Aubrechtová M, Cichowolski M, Fang X, Klug C (2022). Early cephalopod evolution clarified through Bayesian phylogenetic inference. BMC Biology.

[CR102] Ramirez MD, Oakley TH (2015). Eye-independent, light-activated chromatophore expansion (LACE) and expression of phototransduction genes in the skin of *Octopus bimaculoides*. The Journal of Experimental Biology.

[CR103] Reitner J, Engeser T (1982). Phylogenetic trends in phragmocone-bearing coeloids (Belemnomorpha). Neues Jahrbuch Für Geologie Und Paläontologie.

[CR104] Rieber H (1970). *Phragmoteuthis*? *ticinensis* n. sp., ein Coleoidea-Rest aus der Grenzbitumenzone (Mittlere Trias) des Monte San Giorgio (Kt. Tessin, Schweiz). Paläontologische Zeitschrift.

[CR105] Robson GC (1929). On the rare abyssal octopod *Melanoteuthis beebei* (sp. n.): A contribution to the phylogeny of the Octopoda. Proceedings of the Zoological Society of London.

[CR106] Rowe AJ, Kruta I, Landman NH, Villier L, Fernandez V, Rouget I (2022). Exceptional soft-tissue preservation of Jurassic *Vampyronassa rhodanica* provides new insights on the evolution and palaeoecology of vampyroteuthids. Scientific Reports.

[CR107] Rowe AJ, Kruta I, Villier L, Rouget I (2023). A new vampyromorph species from the Middle Jurassic La Voulte-sur-Rhône Lagerstätte. Papers in Palaeontology.

[CR108] Rüppell, E. (1829). Abbildung und Beschreibung einiger neuer oder weniger bekannten Versteinerungen aus der Kalkschieferformation von Solnhofen (p. 12). Brönner: Frankfurt a. M.

[CR109] Sanchez G, Setiamarga DHE, Tuanapaya S, Tongtherm K, Winkelmann IE, Schmidbaur H, Umino T, Albertin C, Allcock L, Perales-Raya C, Gleadall I, Strugnell JM, Simakov O, Nabhitabhata J (2018). Genus-level phylogeny of cephalopods using molecular markers: current status and problematic areas. PeerJ.

[CR110] Sansom RS, Gabbott SE, Purnell ME (2010). Non-random decay of chordate characters causes bias in fossil interpretation. Nature.

[CR111] Schnell AK, Boeckle M, Rivera M, Clayton NS, Hanlon RT (2021). Cuttlefish exert self-control in a delay of gratification task. Proceedings of the Royal Society B.

[CR112] Seilacher A (1970). Begriff und Bedeutung der Fossil-Lagerstätten. Neues Jahrbuch Für Geologie Und Paläontologie, Monatshefte.

[CR113] Shigeno S, Kidokoro H, Tsuchiya K, Segawa S, Yamamoto M (2001). Development of the brain in the oegopsid squid, *Todarodes pacificus*: An atlas up to the hatching stage. Zoological Science.

[CR114] Stubbs AL, Stubbs CW (2016). Spectral discrimination in color blind animals via chromatic aberration and pupil shape. Proceedings of the National Academy of Science u.s.a.

[CR115] Tajika A, Naglik C, Morimoto N, Pascual-Cebrian E, Hennhöfer DK, Klug C (2015). Empirical 3D-model of the conch of the Middle Jurassic ammonite microconch Normannites, its buoyancy, the physical effects of its mature modifications and speculations on their function. Historical Biology.

[CR116] Tanner AR, Fuchs D, Winkelmann IE, Gilbert MTP, Pankey MS, Ribeiro AM, Kocot KM, Halanych KM, Oakley TH, da Fonseca RR, Pisani D, Vinther J (2017). Molecular clocks indicate turnover and diversification of modern coleoid cephalopods during the Mesozoic Marine Revolution. Proceedings of the Royal Society B.

[CR118] Tribble C, Freyman WA, Landis MJ, Lim JY, Barido-Sottani J, Kopperud BT, Höhna S, May MR (2022). RevGadgets: An R package for visualizing Bayesian phylogenetic analyses from RevBayes. Methods in Ecology and Evolution.

[CR119] Trombke, G. (2016). *Morphologische und volumetrische Analysen des Nervensystems und sensorischer Strukturen von* Spirula spirula *Linnaeus, 1758*. 49 pp. BSc thesis, Bonn University.

[CR120] Vannier J, Schoenemann B, Gillot B, Charbonnier S, Clarkson E (2016). Exceptional preservation of eye structure in arthropod visual predators from the Middle Jurassic. Nature Commununications.

[CR121] Vermeij GJ (1977). The Mesozoic marine revolution: Evidence from snails, predators and grazers. Paleobiology.

[CR122] Wani R, Kase T, Shigeta Y, De Ocampo R (2005). New look at ammonoid taphonomy, based on field experiments with modern chambered nautilus. Geology.

[CR123] Ward PD (1979). Cameral liquid in *Nautilus* and ammonites. Paleobiology.

[CR124] Ward PD (1987). The natural history of nautilus.

[CR125] Ward PD, Martin AW (1978). On the buoyancy of the Pearly Nautilus. Journal of Experimental Zoology.

[CR126] Watanabe H, Kubodera T, Moku M, Kawaguchi K (2006). Diel vertical migration of squid in the warm core ring and cold water masses in the transition region of the western North Pacific. Marine Ecology Progress Series.

[CR127] Wells MJ (1963). Taste by touch: Some experiments with *Octopus*. Journal of Experimental Biology.

[CR128] Wells MJ (1964). Detour experiments with Octopuses. Journal of Experimental Biology.

[CR129] Whalen CD, Landman NH (2022). Fossil coleoid cephalopod from the Mississippian Bear Gulch Lagerstätte sheds light on early vampyropod evolution. Nature Communications.

[CR130] Yacobucci MM (2018). Postmortem transport in fossil and modern shelled cephalopods. PeerJ.

[CR131] Young J (1971). The anatomy of the nervous systems of “Octopus Vulgaris”.

[CR132] Zittel, K. A. (1884). *Handbuch der Palaeontologie. I. Abteilung Palaeozoologie. II. Band. Mollusca und Arthropoda*. 958 pp. München, (Oldenbourg).

